# Aroma compounds with enhanced sweet perception in tea infusions: Screening, characterization, and sweetening mechanism

**DOI:** 10.1016/j.jare.2025.05.044

**Published:** 2025-05-20

**Authors:** Yuming Wei, Ya-Ya Yu, Yuan-Chao Li, Xiao-Yu Zhong, Chun Zou, Jingming Ning, Wen-Jiang Dong, Kegang Wu, Yong-Quan Xu

**Affiliations:** a Tea Research Institute Chinese Academy of Agricultural Sciences, National Key Laboratory for Tea Plant Germplasm Innovation and Resource Utilization, Hangzhou 310008, China; b Anhui Agriculture University, National Key Laboratory for Tea Plant Germplasm Innovation and Resource Utilization, Hefei 230036, China; c Zhejiang Minghuang Natural Food Development Co., Hangzhou 310030, China; d Spice and Beverage Research Institute Chinese Academy of Tropical Agricultural Sciences, Wanning 571533, China; e School of Chemical Engineering and Light Industry, Guangdong University of Technology, Guangzhou 510006, China

**Keywords:** Tea infusion, Key aroma compounds, Sweet perception, Aroma-associated sweet, Sweet taste receptor, Molecular docking

## Abstract

**Introduction:**

Tea (*Camellia sinensis*) is globally consumed for its pleasant flavor, with sweetness being a key factor in evaluating tea quality. While taste compounds contribute to this sweetness, aroma also plays a significant role, but its contribution is not well understood in tea infusion.

**Objectives:**

This study aimed to identify aroma compounds that enhance sweetness in tea infusion using a sensomics approach, and explore their synergistic effects through molecular docking.

**Results:**

The aroma increased the sweetness of the tea infusion by more than 24.0 %. Eighteen aroma-active compounds linked to sweetness were identified, among which (*E*)-*β*-damascenone (apple-like), linalool (citrus-like), geraniol (citrus-like), dimethyl sulfide (corn-like), (*E,E*)-2,4-heptadienal (floral), (*E,Z*)-2,6-nonadienal (cucumber-like), (*E*)-linalool oxide (furanoid) (floral), dihydroactinidiolide (fruity), *γ*-nonalactone (coconut-like), and (*E*)-*β*-ionone (floral) had higher sweetness similarity and significantly increased the sweet intensity of sucrose (*p* < 0.05). They likely enhance sweetness by reducing the binding energy of sucrose to sweet taste receptors, forming new hydrogen bonds and hydrophobic interactions.

**Conclusion:**

This study provides new insights into the role of aroma compounds in tea sweetness and suggests a potential mechanism for their sweetening effect. These compounds could be used as flavour enhancers or additives to improve the sweetness of tea beverages.

## Highlight

• The sweet aroma compounds in tea infusion were screened by GC-O-AT.
• Sensomics method explored ten sweetening aroma compounds of tea infusion.
• Sweetening aroma compound-sucrose-sweet receptor was studied by molecular docking.
• Sweetening aroma compounds induced tighter binding of sucrose-sweetness receptors.

## Graphical abstract

**Figure:**
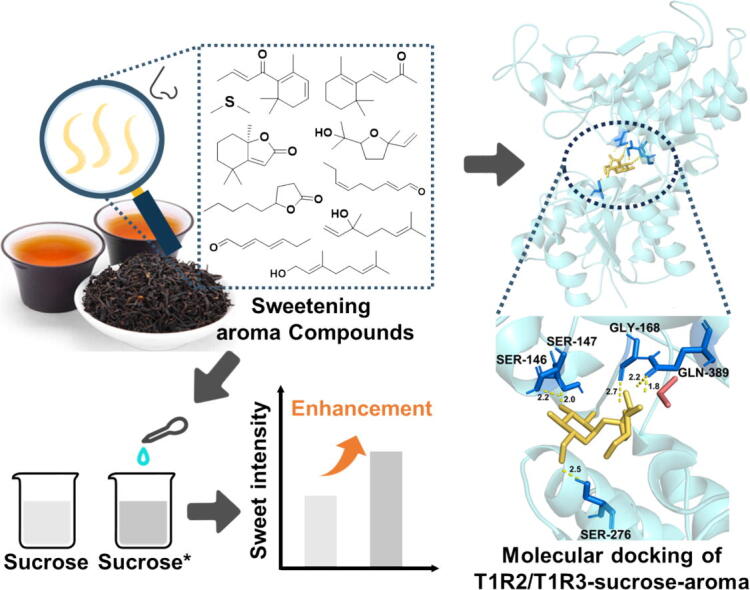

## Introduction

Tea (*Camellia sinensis*), a globally consumed beverage, has garnered significant attention for its pleasant flavor and health benefits [1]. The classification of tea is predicated on various processing methods, yielding six primary categories: green, yellow, white, oolong, black, and dark teas [2]. Notably, the pronounced sweetness in black, white, and yellow teas is a significant attribute that substantially enhances tea infusions’ superior flavor profile [[3], [4], [5]]. Dian black tea, Baimudan white tea, and Huoshan yellow tea are representative examples within their respective categories [[6], [7], [8]].

Sweetness, a natural perceptual effect and a positive evaluation of high-quality tea, forms a richly flavoured tea infusion along with bitterness, astringency and umami [9]. The flavour profile of tea infusion is determined by a multitude of compounds, including caffeine and catechin, which contribute to bitterness and astringency [10]; L-theanine and sucrose, which impart umami and sweetness [9]. Intricate interactions among various compounds influence the sweetness of tea infusions. For instance, epigallocatechin-3-gallate (EGCG) can suppress the sweetness of sucrose, whereas L-theanine exhibits synergistic enhancement properties [11]; malic acid, citric acid, succinic acid, and fumaric acid, which typically associated with sourness, contribute to the sweetness of white tea infusion [12]; Wei et al., (2023) found that phloridzin, dulcitol, and alanine could synergize with sucrose to enhance the sweetness of black tea infusion [3]. However, the contribution of aroma compounds to sweetness perception in tea infusion remains poorly understood.

Aroma-induced sweetness enhancement is an interaction that contributes to developing a high-sweet flavour in food products [13]. When the similarity between aroma and sweetness is substantial, the perception of sweetness is augmented by aroma [14]. Odorants with floral and fruity notes typically exhibit a more remarkable similarity to sweetness and are more prone to exert a sweetness-enhancing effect [15]. Tea is rich in various volatile compounds, but the content is only 0.01 % of the dry weight and is easily affected by the extraction method [16]. Compared with simultaneous distillation extraction (SDE), solid phase micro-extraction (SPME), and solvent-assisted flavour evaporation (SAFE), stir bar sorptive extraction (SBSE) as an environmentally friendly extraction method with high sensitivity and high recovery has been successfully applied to analyse the aroma of tea infusion [17]. Moreover, gas chromatography-olfaction-associated taste (GC-O-AT) analysis can efficiently identify sweetness-related aroma-active compounds in complex food aroma matrices. For example, GC-O-AT was employed to identify sweetness-related aroma compounds in bananas. Sensory evaluations demonstrated that eight aroma-active compounds could increase the sweet intensity of a 30 g/L sucrose solution [18]. Furthermore, Barba et al. (2018) selectively identified sweetness-related aroma compounds using GC-O-AT and discovered that ethyl 2-methylbutanoate, furaneol, and *γ*-decalactone increased sweet intensity in a 7 % sucrose solution [19]. This study integrated sweetness similarity evaluation into the GC-O-AT and sweet intensity evaluation to more accurately identify the key aroma-active compounds in tea infusions that exert a sweetening effect.

The perception of sweetness arises from the interaction of sweet compounds with sweet taste receptors, namely T1R2/T1R3 heterodimers. These receptors are a type of G protein-coupled receptor located in oral taste bud cells [20]. This interaction generates action potentials, releases neurotransmitters, and transmits sweetness signals to the brain’s gustatory orbitofrontal cortex (OFC) [13]. Therefore, an aroma compound’s sweetening effect can be determined by its ability to bind to sweet taste receptors. Molecular docking, a cutting-edge technique in computational chemistry, employs physicochemical principles and algorithms to predict the structural interactions between ligands and receptors. This method optimizes the 3D conformations of protein receptors and small molecule ligands to model their complexes and assesses their binding affinity through binding energy calculations [21]. The objective is to determine the optimal binding conformation for the receptor-ligand interaction, ensuring minimal binding energy and facilitating spontaneous binding [22]. Niu et al. (2024) employed molecular docking to investigate the interactions within the ternary complex comprising sweet taste receptors, aroma compounds, and sucrose and successfully verified that aroma compounds with sweetening effects in sweet oranges were able to form stable complexes with sweet taste receptors-sucrose [23].

Consequently, this study was designed to identify aroma compounds associated with sweetness in three high-sweet teas using SBSE-GC-O-AT analysis. It also aimed to evaluate the sweetness-enhancing effects of these compounds through sensory evaluation and investigate the molecular interactions among sucrose, aroma compounds, and sweet taste receptors using molecular docking. These investigations were conducted to elucidate the potential mechanisms underlying aroma-induced sweetness enhancement in tea infusions. Identifying naturally sweetening aroma compounds in tea infusions offers a novel strategy for sweetening applications in the food industry. Furthermore, this research provides a foundation for future studies on flavour enhancement through aroma-taste interaction.

## Materials and methods

### Tea samples

Three representative tea samples of high-sweet flavour were collected in this study. Black tea samples (BT) were collected from Dian black tea in Yunnan Dianhong Group Co., Ltd. (Fengqing, Yunnan, China); white tea samples (WT) were collected from Baimudan white tea in Fujian Dingbai Tea Co. Ltd. (Fuding, Fujian, China); yellow tea samples (YT) were collected from Huoshan yellow tea in Anhui Huoshan Yellow Tea Group Co. Ltd. (Huoshan, Anhui, China). All samples conform to international standards for Tea — Classification of tea types (ISO 20715–2023). The samples were stored at −20 °C in a freezer for subsequent analysis.

### Chemicals

The C_7_-C_40_ *n*-alkane standard product was purchased from Sigma-Aldrich (Shanghai, China). (*E*)-*β*-Ionone (≥97 %), 2-phenyl-2-butenal (>90 %), 2-heptanol (>90 %), 1-pentanol (≥99.5 %), ethyl caprate (≥99 %), and (*Z*)-3-hexenal (>90 %) were purchased from Macklin Biochemical (Shanghai, China). Linalool (≥96 %) was purchased from TCI Shanghai (Shanghai, China). (*E,Z*)-2,6-Nonadienal (≥95.0 %), (*E,E*)-2,4-Heptadienal (≥5.0 %), *cis*-Jasmone (≥98.0 %), *γ*-nonalactone (≥98.0 %), geraniol (≥98 %), (*E*)-*β*-damascenone (≥98 %) and 2-ethyl-1-hexanol (≥99.5 %) were purchased from Aladdin (Shanghai, China). 3-Methyl-2,4-nonanedione (>90 %) and phenylacetaldehyde (95 %) were purchased from Shanghai Yuanye Bio-Technology Co., Ltd. (Shanghai, China). Dihydroactinidiolide (≥98 %) was purchased from Shanghai Acmec Biochemical Technology Co., Ltd (Shanghai, China). Dimethyl sulfide (≥99 %) and (*E*)-linalool oxide (furanoid) (≥97 %) were purchased from SIGMA-ALDRICH (Beijing, China). All edible-grade aroma compounds were purchased from Shanghai Meixin Chemical Technology Co. Ltd. (Shanghai, China). Sodium chloride (NaCl, ≥99.5 %) was purchased from Shanghai Adamas-Beta Chemical Reagent (Shanghai, China). Sucrose (≥99 %) was purchased from the GCE (Beijing) Technology Co. Ltd (Beijing, China), and drinking water was purchased from Wahaha Group Co. Ltd. (Hangzhou, China).

### Extraction of volatiles by SBSE

The volatiles were extracted via SBSE following a slightly modified [24]. First, a 10 mL aliquot of tea infusion was placed into a headspace vial with 3.0 g of NaCl and ethyl caprate (10 μg/mL as the internal standard). The vial was sealed and equilibrated for 10 min. A polydimethylsiloxane twister (10 mm × 0.5 mm × 24 μL, Gerstel, Germany) was then submerged in the infusion and agitated at 1250 rpm for 60 min at 40 °C. The twister was cleaned, dried, and placed into a thermal desorption tube for GC–MS and GC-O analysis.

### GC–MS analysis

The volatiles were analysed on a gas chromatography (8890A, Agilent, USA) coupled to a mass spectrometer (5977B, Agilent, USA). The volatiles were separated using an HP-INNOWAX column (60 m × 0.25 mm × 0.25 μm, Agilent, USA) with helium as the carrier gas at a flow rate of 1 mL/min. The temperature program involved an initial hold at 40 °C for 5 min, a ramp to 100 °C at 3 °C/min, further to 250 °C at 5 °C/min, and a final hold at 250 °C for 5 min. The mass spectrometer operated in electron ionization mode with an electron energy of 70 eV, an interface temperature of 280 °C, an ion source temperature of 230 °C, and a mass scan range from 33 to 350 *m*/*z*. Identification of the volatiles relied on retention indices (RIs) and mass spectral matching against the NIST20 library. RIs were determined using a series of n-alkanes ranging from C_7_ to C_40_.

### Gas chromatography-olfactometry (GC-O) analysis

A team of five aroma-trained panelists conducted the GC-O analysis. Each panelist assessed the odor characteristics, intensity, and RIs at the sniff port during each chromatographic run. Intensity was rated on a 5-point scale. The odor-specific magnitude estimation (Osme) for each aroma intensity (AI) was determined by averaging the scores from the five panelists. An odorant was considered detectable if identified by at least three of the five panellists [25]. GC-O analysis was conducted on the same gas chromatography, which was fitted with the same mass spectrometer and an olfactory detection port (ODP4, Gerstel, Germany). The volatiles from the capillary column were evenly divided into two streams: one directed to the MS at 250 °C and the other to the sniffing port at 230 °C. The capillary column, gas chromatography, and mass spectrometer conditions were consistent with those used in the GC–MS analysis.

### GC-O-AT analysis

Aroma-trained panelists were tasked with identifying taste-related odors and documenting their taste attributes while also tallying the detection frequency of each taste descriptor (sour, sweet, bitter, salty, and umami) [23]. The RIs for each aroma-active compound were determined by comparing their retention times with those of an *n*-alkane (C_7_ to C_40_) under the same capillary column and gas chromatography conditions.

### Quantitation of aroma-active compounds

The sweetness-related aroma-active compounds were determined using external standard curves (Table S1). Eight solutions of varying concentrations served as aroma standards. GC–MS in selected ion monitoring mode was employed for quantification by measuring the peak areas of the aroma-active compounds relative to specific quantitative ions. The standard curve was constructed by plotting the concentration ratio of the compound to the internal standard on the *x*-axis and the corresponding ratio of peak areas on the *y*-axis [26]. The concentrations of the 18 sweetness-associated aroma-active compounds were determined (Table S1).

### Odor active value (OAV) and aroma character impact (ACI) calculations

OAV is calculated as the concentration of a compound divided by its odor threshold (OT) in water. Compounds with an OAV of 1 or higher contribute significantly to the overall aroma profile [27]. ACI, defined as the ratio of OAVs within a mixture, is determined by the formula provided below [28]:

(1) $${\text{ACI}}_{\text{i}}=\frac{\frac{{\text{P}}_{\text{i}}}{{\text{T}}_{\text{i}}}}{\sum_{\text{k}}\frac{{\text{P}}_{\text{k}}}{{\text{T}}_{\text{k}}}}$$

where P*_i_* denotes the concentration percentage, and T*_i_* denotes the OT of the compound *i*.

### Sensory analysis

#### Ethics statement

All sensory experiments involving humans were conducted according to the ethical policies and procedures approved by the ethics committee of Zhejiang Gongshang University, Hangzhou (Approval no. 24111468). Written consent was obtained from all participants prior to their involvement in the sensory experiments.

#### Recruitment and screening of sensory panelists

Volunteers for sensory testing were recruited from the Tea Research Institute, Chinese Academy of Agricultural Sciences (Hangzhou, China), with criteria excluding smokers, drinkers, and individuals with impaired taste or olfactory senses. According to international standards for Sensory Analysis (ISO 8586-2012), panelists underwent screening and training through taste stimuli identification and intensity rating exercises. A series of five sucrose solutions with varying concentrations (0.1, 0.3, 0.6, 0.9, and 1.2 g/100 mL) was created for sensory volunteers to rank based on perceived sweetness. Only those panelists who achieved a 100 % correct rate in identification and rating tests were deemed qualified for the sensory panel.

#### Training of sensory panelists

Twenty sensory panelists (12 males and eight females, aged 22 to 40 years) were selected and trained using the two-alternative forced choice (2-AFC) method and a 15-point sweet intensity scale. The training was designed to enhance the panelists’ sensitivity to sweetness, ensuring proficiency with both the scale and the 2-AFC method. Panelists evaluated four sucrose solutions of varying concentrations (0.1, 0.2, 0.3, and 0.6 g/100 mL) on the 15-point scale based on sweetness intensity. In the 2-AFC test, panelists were presented with a tea infusion and sucrose solution and tasked with identifying which had the higher perceived sweetness.

#### Descriptive analysis

With minor adaptations from a previous study by Linscott & Lim (2016) [29], initially, the flavour profiles of the tea samples were evaluated using a frequency-based approach. Subsequently, the approach was repeated to evaluate the sweetness-related aroma properties. Aroma compound solutions were prepared according to the specific concentrations for BT, WT, and YT, as listed in Table S1. Each 5 mL sample was served in a 25 mL taste cup with a lid designated by a unique three-digit code. Panelists were required to sip for 5 s and then spit out each sample, describing the retronasal olfactory attributes. To prevent sensory carry-over, they were required to rinse their mouths with ultrapure water after each evaluation.

#### Consistency analysis

Sucrose solutions were prepared at 0.29, 0.11, and 0.18 g/100 mL, corresponding to the relative sweet concentrations of BT, WT, and YT, respectively. These solutions were employed to evaluate the sweetness consistency of aroma compounds. The concentrations listed in Table S1 for sweet-related aroma compounds in tea infusions were employed for consistency analysis. Randomly numbered samples were tasted by the panelists, who were asked to swirl the solution in their mouths for 5 s before rating the perceived sweet similarity. Panelists then used a 10-cm unscaled line scale to rate the aroma compounds, with the left end annotated as “completely different” and the right as “very similar” [29].

#### Sweet intensity evaluation

Five expert panelists with more than five years of sensory evaluation experience were invited to perform preliminary trials using the quantity estimation method. Reference sucrose solutions were utilized at concentrations of 0.10, 0.20, 0.30, and 0.60 g/100 mL, corresponding to sweetness intensity scores of 6, 8, 10, and 15. Initially, the sweet intensity of the tea samples was assessed by 20 panelists, both with and without nose clips. Subsequently, Sucrose solutions were formulated to match the relative sweetness levels of BT, WT, and YT at concentrations of 0.29, 0.11, and 0.18 g/100 mL, respectively. These solutions were employed to evaluate the sweetness-enhancing impact of aroma compounds (Table S2). The sweet intensity of the aroma-sucrose mixture samples was evaluated without nose clips. The sweetness intensity was scored on a 15-point scale, ranging from “no perception” at 1 to “most intense” at 15[29].

### Molecular docking

For molecular docking analysis, ten sweetening aroma compounds were selected as ligands. The structural template for the human sweet taste receptor, consisting of T1R2/T1R3, was derived from Chéron et al. (2017) [30]. We focused on the venus-flytrap-like domains (VFD) of T1R2/T1R3 as the principal binding regions and created a 3D grid with an exhaustiveness parameter set to 8. Aroma compound 3D models were generated using ChemDraw 20.0 (PerkinElmer, USA). Docking simulations were conducted with AutoDockTools (ADT, V1.5.7, Scripps Research, USA), and the results were visualized in PyMOL (DeLano Scientific LLC, USA). Each aroma ligand was docked individually to the sweet taste receptors. Following this, sucrose was docked with the receptors to form a receptor-ligand complex, which was then used for semi-flexible docking with the aroma ligands [31].

### Statistical analysis

All experiments were replicated a minimum of three times. Statistical analysis of the experiment results was conducted via one-way analysis of variance (ANOVA) at p < 0.05. For sweet similarity and intensity data, ANOVA was supplemented with Duncan’s multiple range test to identify significant differences at *p* < 0.05. SPSS Statistics 24.0 software (IBM Inc., Chicago, USA) was utilized for all analyses.

## Result

### Flavor characteristics and volatiles profile of three tea infusions

**Fig. 1:**
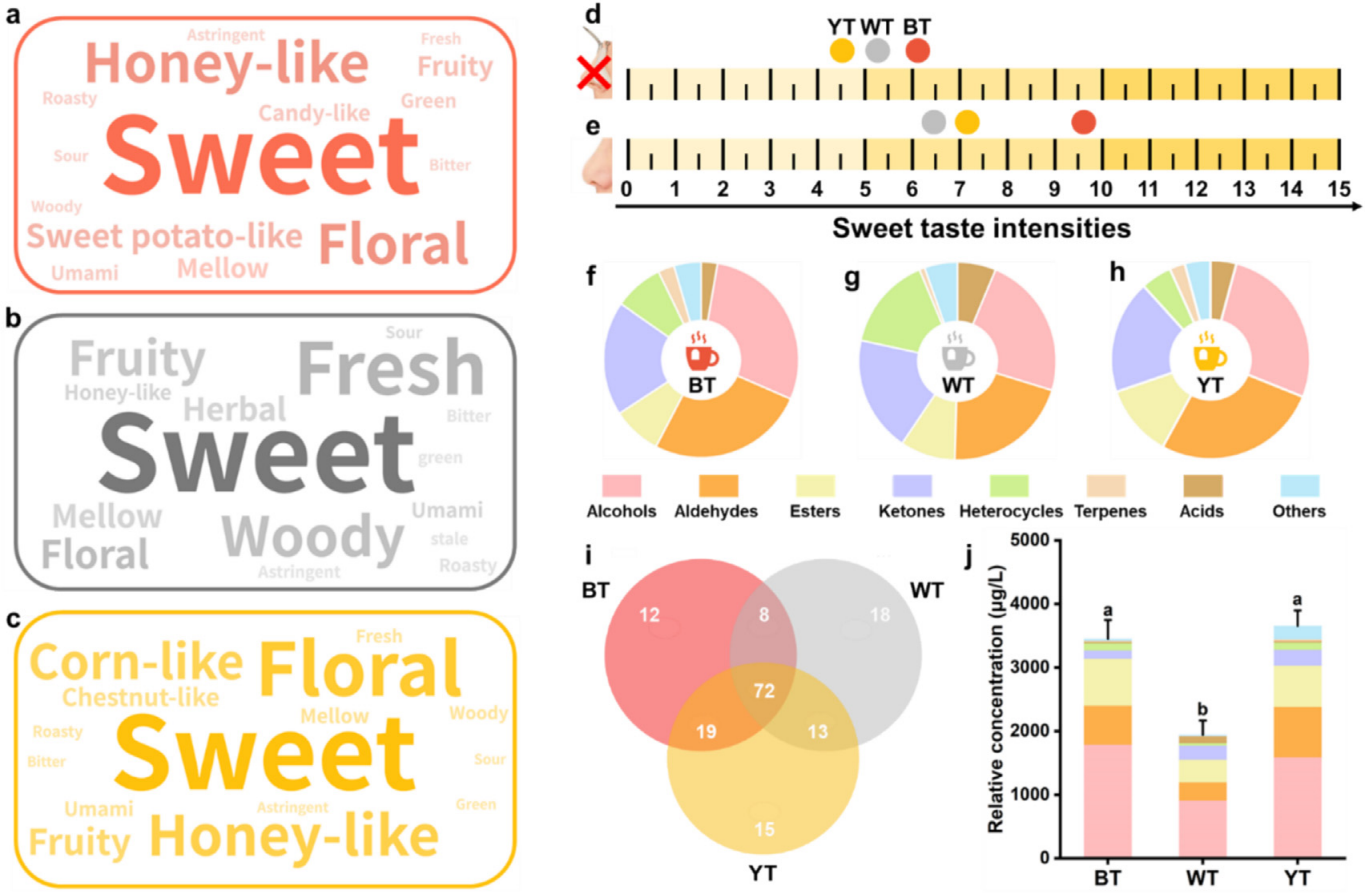
**Flavour profiles of three tea infusions.** Flavour word clouds of BT (a), WT (b), and YT (c); Sweetness intensity of BT, WT, and YT with (d) and without nose clips (f); Percentage of volatile compound classes of BT (f), WT (g), and YT (h); Venn of volatile compounds in three tea infusions (i); relative concentrations of volatile compounds in three tea infusions (j). BT indicates black tea; WT indicates white tea; YT indicates yellow tea. Different letters indicate significant differences at the *p* < 0.05.

As shown in Fig. 1, the flavor profiles of the tea samples were analyzed, and the highest word frequency of “sweet” was found in three tea samples (BT, WT, and YT). Meanwhile, high word frequencies of “honey-like” and “floral” were found in BT (Fig. 1a); high word frequencies of “fresh,” “woody,” and “fruity” was found in WT (Fig. 1b); high word frequencies of “floral”, “corn-like” and “honey-like” were found in YT (Fig. 1c).

To explore the effect of these flavor profiles on sweet, we evaluated the sweet intensity of the BT, WT, and YT using nose clips (Fig. 1d) with and without nose clips (Fig. 1e). The results showed that the sweet intensity of the three tea samples without nose clips was significantly increased, with BT sweetness increased by 59.8 %, WT sweetness increased by 24.0 %, and YT sweetness increased by 56.0 %.

Therefore, we further analysed the volatiles in BT, WT, and YT using SBSE-GC–MS. 157 volatiles were identified, including 39 aldehydes, 38 alcohols, 25 ketones, 20 heterocyclic, 16 esters, eight acids, three terpenes, and eight others (Table S3). As shown in Fig. 1f, g, and h, the BT, WT, and YT volatiles were dominated by alcohols, aldehydes, and ketones. And according to Fig. 1i and j, BT and YT had richer and higher relative volatile concentrations than the WT. This result is consistent with the effect of aroma on sweet intensity enhancement in Fig. 1d and e, suggesting that aroma compounds with enhancing sweet intensity might be more abundant and concentrated in BT and YT. However, not all volatiles are aroma-active compounds, so it is first necessary to find aroma-active compounds among these volatiles [5].

### Potential sweet-related aroma-active compounds in tea infusions

The GC-O technique is a reliable method for detecting aroma-active compounds within tea infusions [32]. GC-O-AT analysis was employed to identify potential sweet-related aroma-active compounds and the sweetness of the tea infusion to validate the link between GC–MS results and sensory data and determine the correlation between aroma-active compounds [23,25].

#### GC-O analysis of the aroma characteristics in tea infusions

**Fig. 2:**
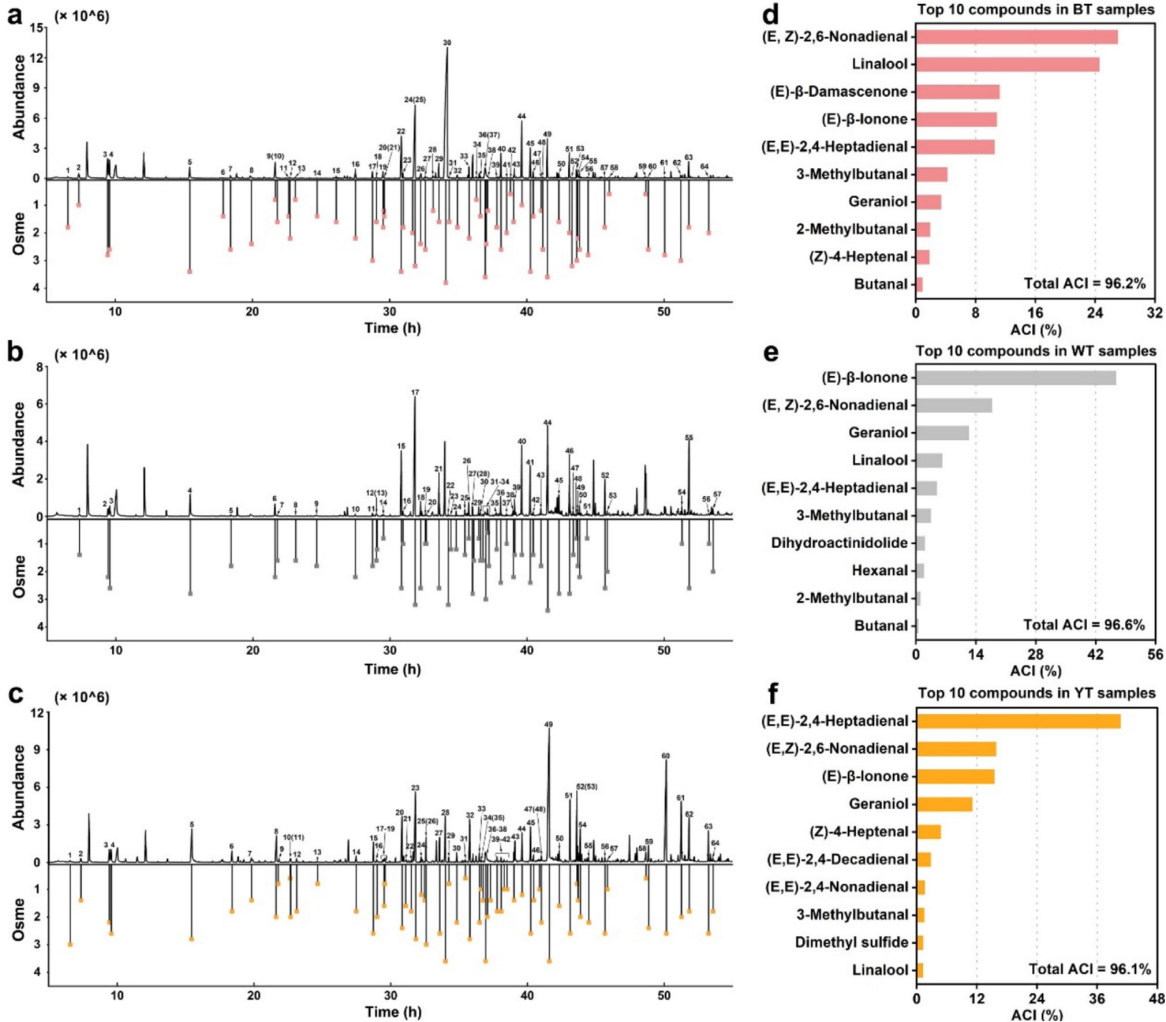
**Analysis of aroma-active compounds in three tea infusions.** GC-O chromatogram and corresponding odor specific magnitude estimation (Osme) of BT (a), WT (b), and YT (c); aroma character impact (ACI) value top 10 aroma-active compounds in BT (d), WT (e), and YT (f). BT indicates black tea; WT indicates white tea; YT indicates yellow tea.

A total of 74 odorants were identified in BT, WT, and YT by SBSE-GC-O (Table S3). Among them, 64 odorants were sniffed and identified in BT (Fig. 2a), 57 odorants were sniffed and identified in WT (Fig. 2b), and 64 odorants were sniffed and identified in YT (Fig. 2c). The aroma intensity (AI) and attributes of aroma-active compounds are key factors affecting the flavor quality of tea infusion [25]. We analyzed the odor attributes and intensity of the aroma-active compounds sniffed by GC-O. As shown in Table S3, the AIs of the aroma-active compounds with floral and fruity flavors in the three tea infusions generally had high values. For example, linalool (citrus-like, AI = 3.8) had the highest odor intensity, followed by phenylacetaldehyde (honey-like, AI = 3.6) and geraniol (citrus-like, AI = 3.6) in BT infusion; geraniol (citrus-like, AI = 3.4) had the highest odor intensity, followed by linalool (citrus-like, AI = 3.2) and (*E*)-linalool oxide (furanoid) (floral, AI = 3.2) in WT infusion; geraniol (citrus-like, AI = 3.6), linalool (citrus-like, AI = 3.6), and phenylacetaldehyde (honey-like, AI = 3.6) had the highest odor intensity, followed by (*E,E*)-2,4-heptadienal (floral, AI = 3.0) and dimethyl sulfide (corn-like, AI = 3.0) in YT infusion.

We calculated their ACI values to assess the role of high-odor-intensity aroma-active compounds in shaping the tea’s flavour profile. These values reflect the olfactory impact of aroma compounds and are determined by their relative concentrations [1]. Those compounds with higher ACI values are deemed to have a more substantial effect on the aroma of tea [28]. The top ten aroma-active compounds with the highest ACI values in BT, WT, and YT were listed in Fig. 2d, e, and f, respectively. The results showed that the total ACIs of the top ten compounds in the three tea samples exceeded 95 %, indicating that they contributed significantly to forming the aroma of the three tea infusions. In BT, the aroma-active compound with the highest ACI value was (*E,Z*)-2,6-nonadienal (cucumber-like, ACI = 27.0 %), followed by linalool (ACI = 24.5 %), (*E*)-*β*-damascenone (apple-like, ACI = 11.2 %), (*E*)-*β*-ionone (floral, ACI = 10.8 %) and (*E,E*)-2,4-heptadienal (floral, ACI = 10.5 %). In WT, the aroma-active compound with the highest ACI value was (*E*)-*β*-ionone (ACI = 46.7 %), followed by (*E,Z*)-2,6-nonadienal (ACI = 17.8 %), geraniol (ACI = 12.3 %), linalool (ACI = 6.1 %) and (*E,E*)-2,4-heptadienal (ACI = 4.9 %). In YT, the aroma-active compound with the highest ACI value was (*E,E*)-2,4-heptadienal (ACI = 40.7 %), followed by (*E,Z*)-2,6-nonadienal (ACI = 15.8 %), (*E*)-*β*-ionone (ACI = 15.5 %), geraniol (ACI = 11.1 %) and (*Z*)-4-heptenal (oily and fatty, ACI = 4.8 %). These results showed that the aroma-active compounds with a higher percentage of ACI were predominantly characterized by floral and fruity flavours, which are often associated with sweetness [15]. Therefore, we further analysed the taste attributes of these aroma-active compounds using GC-O-AT to find potential sweet-related aroma-active compounds.

#### GC-O-AT analysis to find sweet-related aroma-active compounds

A total of 18 aroma-active compounds associated with sweetness were identified by GC-O-AT (Table 1). These compounds included alcohols, aldehydes, and ketones. Most of them exhibited floral and fruity notes, in accord with the known associations between odor profiles and sweetness perception [15]. The result showed that the number of aroma-active compounds associated with sweetness was highest in BT, followed by YT, and lowest in WT. More than 80 % of the panelists considered these aroma-active compounds, such as (*E*)-*β*-damascenone, *γ*-nonalactone, linalool, (*E*)-linalool oxide (furanoid), 3-methyl-2,4-nonanedione, and geraniol, to be related to sweetness due to their fruity, coconut-like, floral, and honey-like aromas. Among them, (*E*)-*β*-damascenone, linalool, and geraniol significantly enhanced aroma and sweetness in reported flavour studies of osmanthus-scented green tea and brown sugar [31,33]. Moreover, most of these sweet-related aroma-active compounds were also the key compounds with high ACI values in BT, WT, and YT (Fig. 2d, e, and f). This result further suggests that these key aroma-active compounds may function in the perception of sweetness while forming the characteristic aroma.

**Table 1:** The sweet-related aroma-active compounds were identified by GC-O-AT.

No.	RI^a^	CAS	Aroma-active compounds	Odor description^b^	Taste association^c^	
					Main	Others
1	817	75-18-3	Dimethyl sulfide	corn-like	sweet 58.3 %	sour 8.3 %
2	1154	6789-80-6	(*Z*)-3-Hexenal	green, grassy	sweet 41.7 %	bitter 25.0 %
3	1264	71-41-0	1-Pentanol	fruity, ethereal	sweet 75.0 %	−
4	1328	543-49-7	2-Heptanol	coconut-like	sweet 25.0 %	−
5	1479	34995-77-2	(*E*)-Linalool oxide (furanoid)	floral	sweet 83.3 %	−
6	1490	104-76-7	2-Ethyl-1-hexanol	ethereal, fruity	sweet 75.0 %	umami 41.7 %
7	1499	4313-03-5	(*E*,*E*)-2,4-Heptadienal	fatty, floral	sweet 66.7 %	Salty 16.7 %
8	1556	78-70-6	Linalool	citrus-like, floral	sweet 91.7 %	−
9	1594	557-48-2	(*E*,*Z*)-2,6-Nonadienal	cucumber-like	sweet 66.7 %	umami 33.3 %
10	1657	122-78-1	Phenylacetaldehyde	floral, honey-like	sweet 58.3 %	−
11	1730	113486-29-6	3-Methyl-2,4-nonanedione	rose-like, fruity	sweet 83.3 %	−
12	1833	23726-93-4	(*E*)-*β*-Damascenone	apple-like	sweet 100.0 %	−
13	1849	106-24-1	Geraniol	rose-like, citrus-like	sweet 83.3 %	sour 16.7 %
14	1948	4411-89-6	2-Phenyl-2-butenal	cocoa-like,	sweet 75.0 %	bitter 16.7 %
15	1952	79-77-6	(*E*)-*β*-Ionone	floral, violet-like	sweet 66.7 %	bitter 8.3 %
16	1959	488-10-8	*cis*-Jasmone	woody, herbal, floral	sweet 41.7 %	bitter 25.0 %
17	2049	104-61-0	*γ*-Nonalactone	coconut-like	sweet 100.0 %	umami 41.7 %
18	2388	17092-92-1	Dihydroactinidolide	fruit, woody	sweet 50.0 %	−

a Retention index (RI) was calculated by HP-INNOWAX capillary columns;

b Odor description of each odorant detected at the olfactory detection port;

c Frequency of taste descriptions of aroma-active compounds.

### Similarity of aroma and sweet perception of aroma-active compounds

**Fig. 3:**
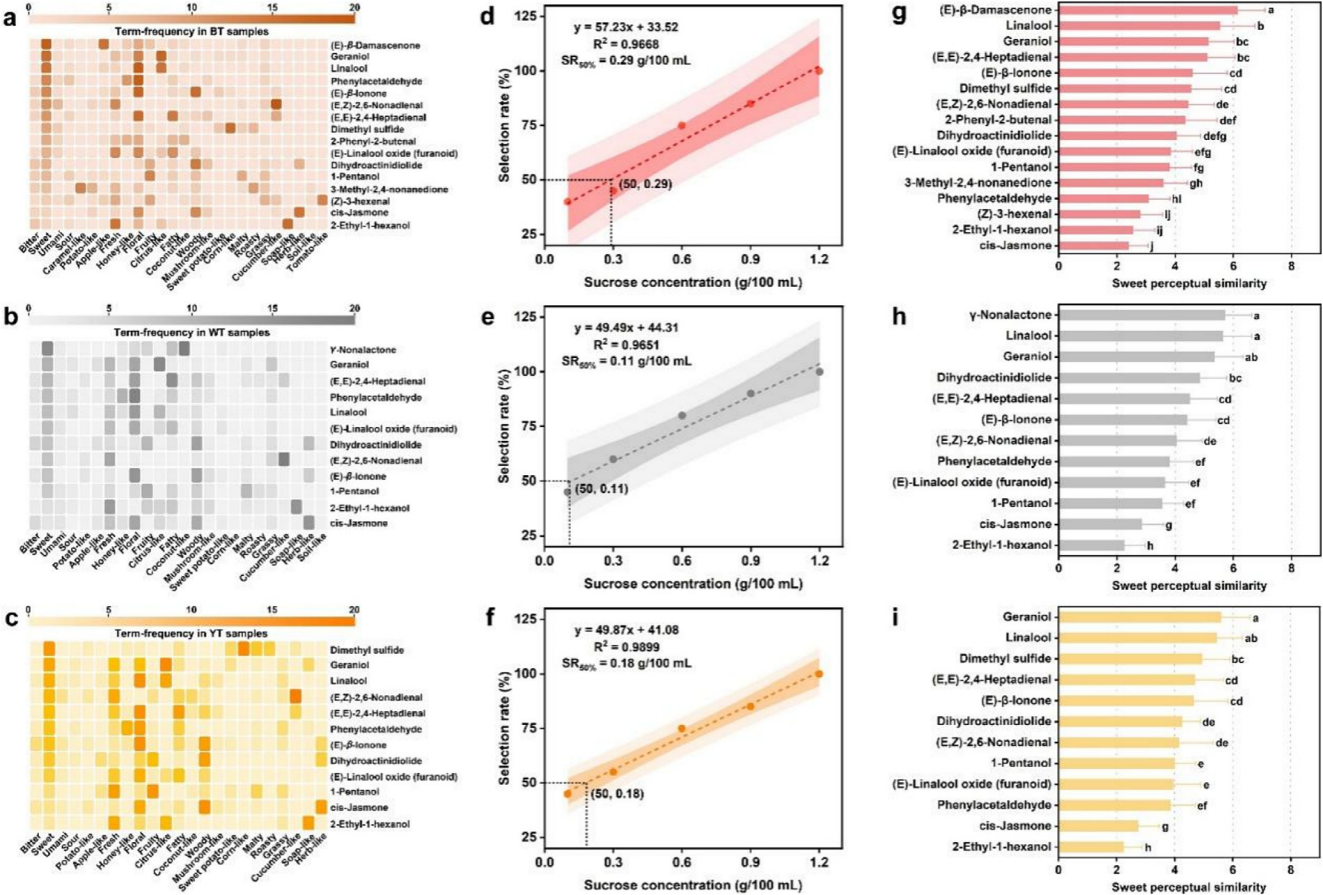
**Analysis of the similarity between aroma-active compounds and sweetness in three tea infusions.** Heat map of the frequency of flavour characterization of aroma-active compounds associated with sweetness in BT (a), WT (b), and YT (c); the sucrose concentration-selection rate (C-SR) regression curve of BT (d), WT (e), and YT (f); the similarity rating of sweet perception for the amora-active compounds associated with sweetness in BT (g), WT (h), and YT (i). BT indicates black tea; WT indicates white tea; YT indicates yellow tea. Different letters indicate significant differences at the *p* < 0.05.

These 18 sweetness-related aroma-active compounds were quantified using external standards and calculated OAV (Table S1). Compounds with an OAV of 1 or higher were identified as contributors to the tea infusion’s aroma profile [34]. The results showed that 17 aroma-active compounds (except for 2-heptanol) had an OAV greater than 1, including 16 in BT, 12 in WT, and 12 in YT (Table S1). Therefore, the flavour descriptive analysis of the 17 aroma-active compounds was performed. Twenty-eight flavour descriptors were generated, including 26 for BT, 24 for WT, and 23 for YT. Fig. 3a, b, and c show each aroma-active compound’s flavour descriptors and word frequencies.

(*E*)-*β*-damascenone, which is unique in BT, mainly presented sweet, apple-like, and floral flavors, and the word frequency of “sweet” (95 %) was the highest in BT (Fig. 3a), which is consistent with the results of GC-O-AT, suggesting that (*E*)-*β*-damascenone might have an important contribution to the enhancement of sweet in BT infusion. In WT, the unique *γ*-nonalactone presented sweet, coconut-like, and fatty flavors, of which “coconut-like” (100 %) and “sweet” (90 %) had the highest word frequencies (Fig. 3b). In the GC-O-AT results, all panelists associated *γ*-nonalactone with the perception of sweetness, probably caused by the coconut-like flavour. We speculated that *γ*-nonalactone might be the key aroma-active compound that causes the enhanced sweetness perception of WT. Compared to BT, dimethyl sulfide with a corn-like flavor showed a higher word frequency of “sweet” in YT (Fig. 3c). Based on the results of the external standard quantification (Table S1), it can be found that the concentration of dimethyl sulfide in YT was higher than that in BT, which indicates that the panelists might have a more significant sweet perception for the relatively high concentration of dimethyl sulfide. Linalool, geraniol, phenylacetaldehyde, (*E,E*)-2,4-heptadienal, (*E,Z*)-2,6-nonadienal, dihydroactinidiolide, and (*E*)-*β*-ionone were described as having floral, citrus-like, honey-like, cucumber-like, or woody flavors in BT, WT, and YT. These aroma compounds all have a particular sweet flavour. However, we found that 2-ethyl-1-hexanol and *cis*-jasmone, which were associated with sweetness in GC-O-AT, did not have a high frequency of “sweet” flavour descriptors. The variation could stem from disparities in orthonasal versus retronasal olfactory perception. Research indicates that identical aroma compounds can trigger distinct neural reactions through orthonasal and retronasal olfactory pathways, resulting in divergent flavour sensations [35].

To further investigate the relationship between odor and sweetness of these 17 aroma-active compounds, sucrose concentration-selection rate (C-SR) regression curves were derived for three tea infusions using the 2-AFC method, with R2 of 0.9668 for BT (Fig. 3d), 0.9651 for WT (Fig. 3e), and 0.9899 for YT (Fig. 3f), indicating that the linear equation fits well. The relative sucrose concentrations for BT, WT, and YT were calculated based on the 50 % selection rates of 0.29 g/100 mL, 0.11 g/100 mL, and 0.18 g/100 mL, respectively. The three sucrose solutions (0.29 g/100 mL, 0.11 g/100 mL, and 0.18 g/100 mL) were used as references to score the similarity between their odors and sweetness. (*E*)-*β*-Damascenone had the highest odor and sweetness similarity scores (6.2) in BT (Fig. 3g); *γ*-nonalactone had the highest odor and sweetness similarity scores (5.7) in WT (Fig. 3h); and geraniol had the highest odor and sweetness similarity scores (5.6) in YT (Fig. 3i). Meanwhile, linalool, dimethyl sulfide, and (*E,E*)-2,4-heptadienal had odor and sweetness similarity scores of more than 5 in BT, WT, or YT, suggesting that the odors of these compounds may be able to produce a perception of sweetness. However, not all odors produce the perceived sweetness without any taste compounds. For example, the odor and sweetness similarity scores for *cis*-jasmone and 2-ethyl-1-hexanol, which were present in BT, WT, and YT, were below 3, and the panelists generally felt that the strong herb-like and soap-like odors limited sweetness perception. This result suggests that the olfactory perception of odorants plays a significant role in shaping the intensity of sweetness perception [13].

### Effect of key aroma-active compounds on the sweet intensity

**Fig. 4:**
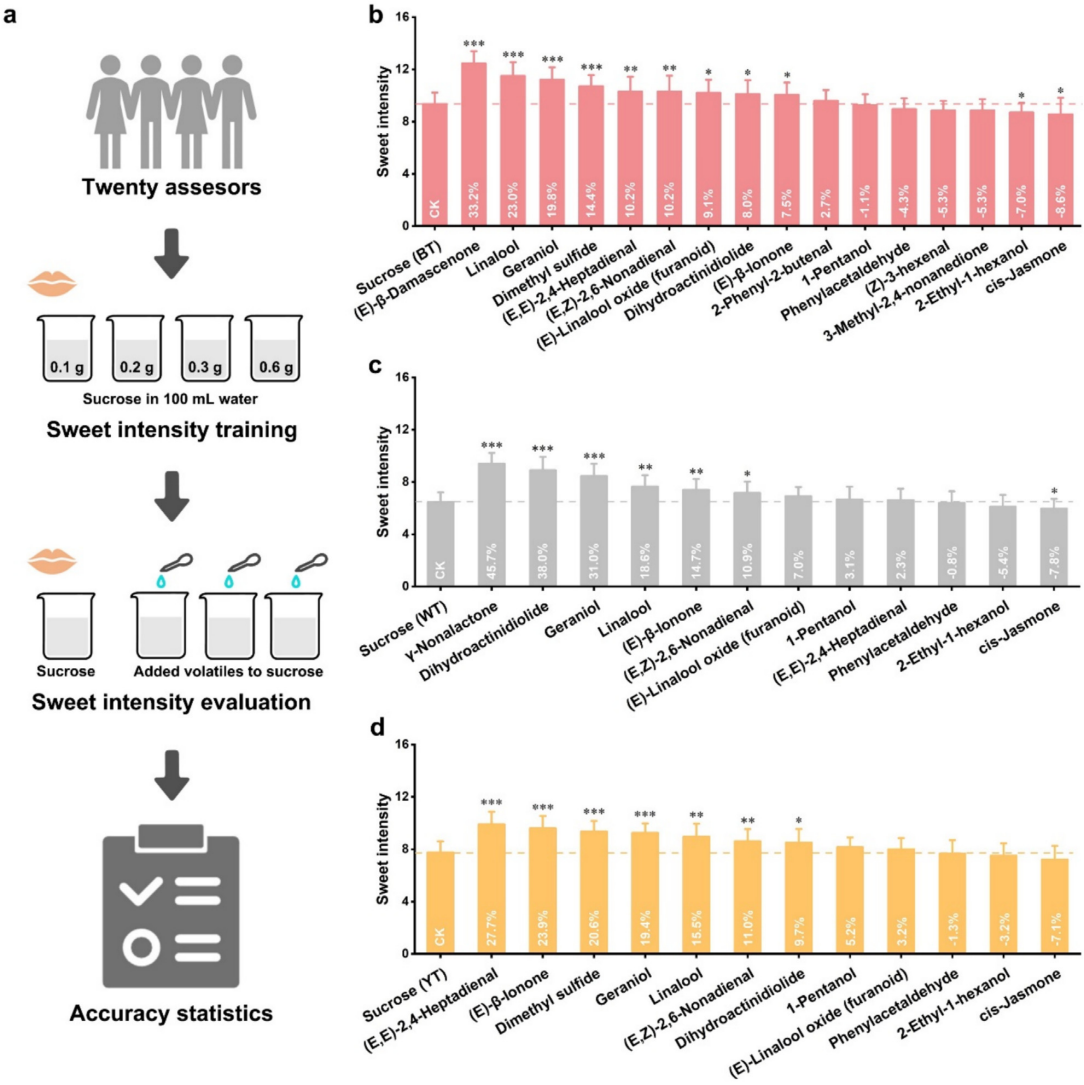
**Effect of aroma-active compounds similar to sweetness in three tea infusion on sucrose sweetness.** Flow chart of the sweet intensity evaluation (a). The sweet intensity rating for sucrose solution with/without aroma compound of BT concentration level (b), WT concentration level (c), and YT concentration level (d). BT indicates black tea; WT indicates white tea; YT indicates yellow tea. *** indicates significance at *p* < 0.001, ** indicates significance at *p* < 0.01, and * indicates significance at *p* < 0.05.

In the sweetness evaluation section (Fig. 4a), the panelists trained in sweet intensity were required to rate the sweet intensity of three sucrose solutions (0.29 g/100 mL, 0.11 g/100 mL, and 0.18 g/100 mL) to which sweet-related aroma-active compounds had been added (Table S3). Sucrose solutions without added odorants were used as controls. Fig. 4b, c, and d showed the enhancing effect of these sweet-related aroma-active compounds on sweet intensity.

In BT, (*E*)-*β*-damascenone, linalool, geraniol, dimethyl sulfide, (*E,E*)-2,4-heptadienal, (*E,Z*)-2,6-nonadienal, (*E*)-linalool oxide (furanoid), dihydroactinidiolide, and (*E)-β*-ionone had significant sweetening effects (*p* < 0.05). Notably, (*E*)-*β*-damascenone, which had an “apple-like” and “sweet” aroma and the highest similarity to sweetness in BT, showed the most significant enhanced sweetness effect. The enhanced sweetness effect of (*E*)-*β*-damascenone is likely due to its “apple-like” and “sweet” aroma and high similarity to sweetness. This aroma, entering the olfactory system via the retronasal pathway, concurrently affects taste sensations in the oral cavity. The resulting cross-modal interaction elicits a distinctive and lasting sweetness and feelings of pleasure and relaxation [23].

In WT, *γ*-nonalactone, dihydroactinidiolide, geraniol, linalool, (*E*)*-β*-ionone, and (*E,Z*)-2,6-nonadienal had significant sweetening effects (*p* < 0.05). Among them, *γ*-nonalactone with a “coconut-like” aroma showed WT’s most significant sweetening effect. *γ*-Nonalactone also showed high sweetness similarity, which confirms that the higher the similarity between odor and sweetness, the more likely it is to have a sweetening effect [15].

In YT, (*E,E*)-2,4-heptadienal, (*E*)-*β*-ionone, dimethyl sulfide, geraniol, linalool, (*E,Z*)-2,6-nonadienal, and dihydroactinidiolide showed significant sweetening effects (*p* < 0.05). Of these, (*E,E*)-2,4-heptadienal with a “floral” aroma had the most significant sweetening effect in YT. The inclusion of aroma compounds with “sweet” or “sweet-congruent” characteristics in a solution can intensify its sweetness, resulting in a heightened sweet perception [36].

Although all the aroma compounds screened were associated with sweetness, not all had a sweetening effect [23]. For example, *cis*-jasmone and 2-ethyl-1-hexanol showed no sweetening effect and even significantly reduced sweetness intensity (*p* < 0.05). Most panelists believe these two odorants have unpleasant “herbal” and “soapy” odors, lowering acceptability and inhibiting sweetness perception.

In summary, the aroma-active compounds with significant sweetening effects on the three highly sweetened tea infusions mainly included (*E*)-*β*-damascenone, (*E*)-*β*-ionone, geraniol, linalool, (*E*)-linalool oxide (furanoid), *γ*-nonalactone, (*E,E*)-2,4-heptadienal, dimethyl sulfide, (*E,Z*)-2,6-nonadienal, and dihydroactinidiolide. This sweetening effect is strongly influenced by the structure and aroma type of the compound [37]. The structure of aroma compounds determines their interaction with taste receptors, while the type of aroma can modulate the overall flavour experience [23]. Understanding these complex interactions is crucial for developing food products and flavourings that can optimize sweetness perception through aroma [13].

### Molecular docking of sweetening aroma compounds to sweet taste receptor-sucrose complexes

**Fig. 5:**
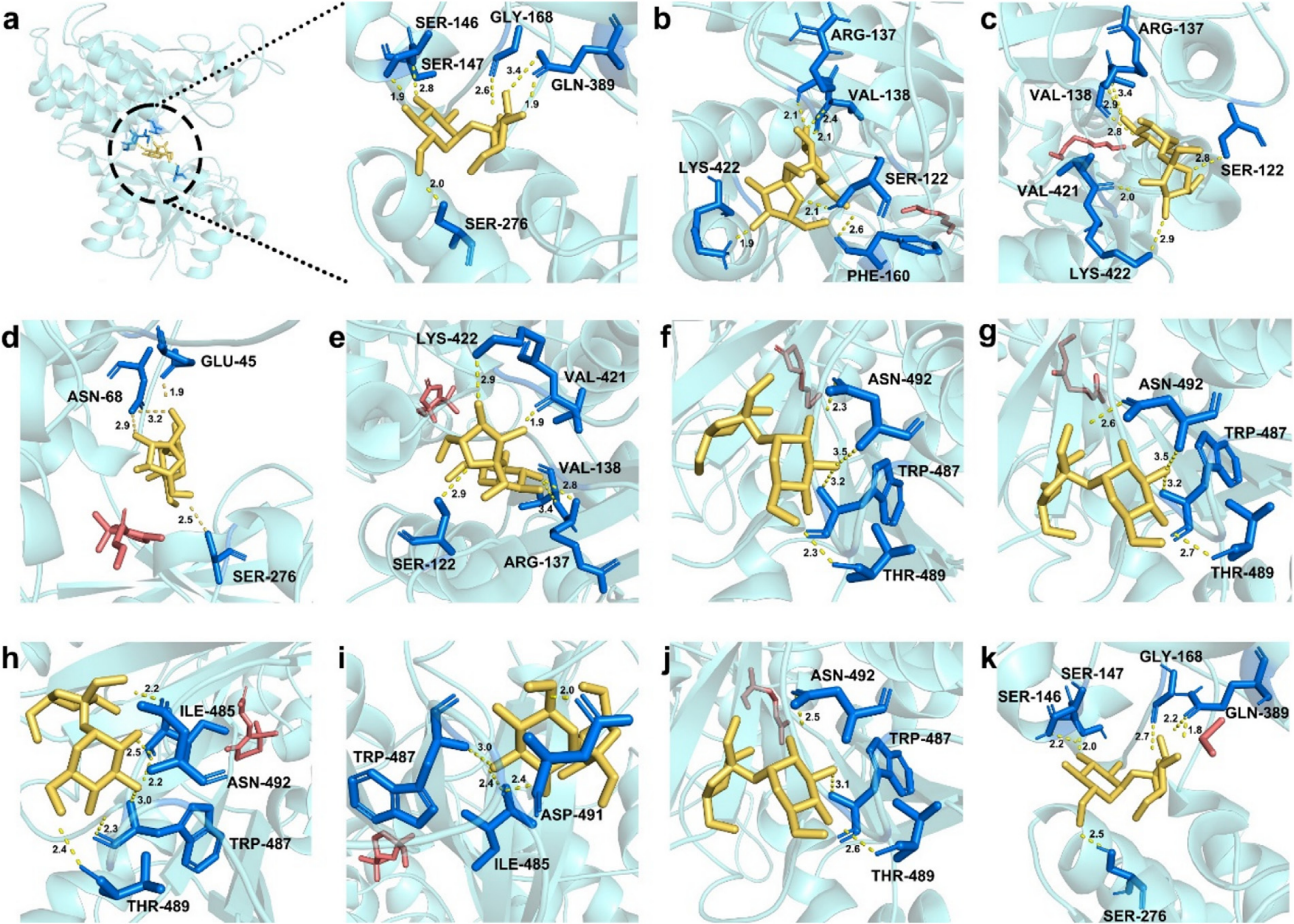
**Effect of sweetening aroma compounds on the binding capacity of sucrose to sweet taste receptors.** 3D docking diagram of T1R2/T1R3-VFD protein and sucrose molecule (a). the 3D interaction diagrams of sucrose with (*E,E*)-2,4-heptadienal (b), (*E,Z*)-2,6-nonadienal (c), (*E*)-*β*-damascenone (d), dihydroactinidiolide (e), *γ*-nonalactone (f), geraniol (g), (*E*)-*β*-ionone (h), (*E*)-linalool oxide (furanoid) (i), linalool (j), dimethyl sulfide (k) and sweet taste receptors respectively. Yellow represents sucrose, red represents aroma compounds, and blue represents amino acid residues that sucrose acts on.

To probe the interaction mechanisms between aroma compounds and sweet taste receptors and clarify the aroma-induced sweetening effect, initial binary complexes were created with aroma-active compounds with a notable sweetening impact and sweet taste receptors. Following this, sucrose was incorporated into these complexes to yield ternary complexes that included sucrose, aroma compounds, and sweet taste receptors [23]. This approach was used to assess the effect of aroma compounds on sucrose binding. As depicted in Fig. 5a and Fig. S1, the interaction is confined to the VFD of the T1R2/T1R3.

We selected the conformations that exhibited the lowest binding free energies from the docking results, as shown in Fig. 5. Negative binding energy signifies the spontaneous nature of molecular binding interactions [23]; the smaller the binding energy, the easier it is to undergo spontaneous binding and the more stable the spontaneous binding structure [38]. As shown in Table 2, the minimum binding energy of sucrose to the sweet taste receptor decreases with the addition of aroma compounds with sweetening effects. The binding energies post-interaction for each aroma compound were as follows: (*E,E*)-2,4-heptadienal (−5.9 kcal/mol), (*E,Z*)-2,6-nonadienal (−6.0 kcal/mol), (*E*)-*β*-damascenone (−5.4 kcal/mol), dihydroactinidiolide (−6.0 kcal/mol), *γ*-nonalactone (−5.4 kcal/mol), geraniol (−5.4 kcal/mol), (*E*)-*β*-ionone (−5.6 kcal/mol), (*E*)-linalool oxide (furanoid) (−5.5 kcal/mol), linalool (−5.4 kcal/mol) and dimethyl sulfide (−6.3 kcal/mol). In contrast, the minimum binding energy of sucrose to the binary molecular docking system of the sweet taste receptor was −5.2 kcal/mol. This result was consistent with the results found by Acevedo et al. (2018), who suggested that the minimum binding energy of sweet compounds to sweet taste receptors was negatively correlated with sweetness [39]. This phenomenon could be attributed to the influence of aroma compounds with sweetening effects on the binding region, site, and conformational arrangement of the T1R2/T1R3-sucrose complex, which in turn impacts the recognition and binding affinity of T1R2/T1R3 for sucrose [23].

**Table 2:** Minimum binding free energy of T1R2/T1R3- aroma compounds with sucrose, amino acids bonded by hydrogen bonds, and amino acids that produce hydrophobic interactions.

Receptors and ligands	Minimum binding energy (kcal/mol)	Hydrogen bond site	Hydrophobic interaction site
T1R2/T1R3-sucrose	−5.2	Ser146, Ser147, Gly168, Ser276, Gln389	−
T1R2/T1R3-(*E*,*E*)-2,4-heptadienal-sucrose	−5.9	Ser122, Arg137, Val138, Phe160, Lys422	Ser147, Tyr218, Glu301, Ala302
T1R2/T1R3-(*E*,*Z*)-2,6-nonadienal-sucrose	−6.0	Ser122, Arg137, Val138, Val421, Lys422	Ser147, Tyr218, Glu301, Ala302
T1R2/T1R3-(*E*)-*β*-damascenone- sucrose	−5.4	Glu45, Asn68, Ser276	Asp216, Glu217, Tyr218, Ala302
T1R2/T1R3-dihydroactinidiolide-sucrose	−6.0	Ser122, Arg137, Val138, Val421, Lys422	Ser170, Met171, Tyr218
T1R2/T1R3-*γ*-nonalactone-sucrose	−5.4	Trp487, Thr489, Asn492	Try218, Ala302, His388, Gln389
T1R2/T1R3-geraniol-sucrose	−5.4	Trp487, Thr489, Asn492	Ser170, Try218, Glu301, Ala302
T1R2/T1R3-(*E*)-*β*-ionone-sucrose	−5.6	Ile485, Trp487, Thr-489, Asn492	Tyr218, Glu301, Ala302
T1R2/T1R3-(*E*)-linalool oxide (furanoid)-sucrose	−5.5	Ile485, Trp487, Asp491	Ser147, Ser170, Tyr218, Glu301
T1R2/T1R3-linalool-sucrose	−5.4	Trp487, Thr-489, Asn492	Gly168, Tyr218, Glu301, Ala302
T1R2/T1R3-dimethyl sulfide-sucrose	−6.3	Ser146, Ser147, Gly168, Ser276, Gln389	−

Throughout the docking process, protein–ligand interactions are predominantly of two kinds: hydrogen bonds and hydrophobic interactions. Fig. 5b-k identifies the critical residues that participate in hydrogen bonding during the ternary docking of sucrose (in yellow) and sweetening aroma compounds (in red) with the T1R2/T1R3 receptors. The addition of these ten sweetening aroma compounds facilitates the formation of hydrogen bonds between sucrose and sweet taste receptors, exemplified by the interactions of (*E,E*)-2,4-heptadienal with Ser122, Arg137, Val138, Phe160, and Lys422; (*E,Z*)-2,6-nonadienal with Ser122, Arg137, Val138, Val421, and Lys422; (*E*)-*β*-damascenone with Glu45, Asn68, and Ser276; dihydroactinidiolide with Ser122, Arg137, Val138, Val421, and Lys422; *γ*-nonalactone with Trp487, Thr489, and Asn492; geraniol with Trp487, Thr489, and Asn492; (*E*)-*β*-ionone with Ile485, Trp487, Thr489, and Asn492; (*E*)-linalool oxide (furanoid) with Ile485, Trp487, and Asp491; linalool with Trp487, Thr489, Asn492; and dimethyl sulfide with Ser146, Ser147, Gly168, Ser276, and Gln389. Oxygen-containing functional groups in sucrose render it prone to engage with amino acid residues, such as amines and carboxyl groups, emphasizing the significance of hydrogen bonding in ligand-receptor binding [23]. In addition, hydrophobic interactions are also observed between the receptor and the ligand. Key hydrophobic residues identified in this study are Tyr218, Glu301, and Ala302, which are nearby, facilitating the formation of a coherent hydrophobic zone. From Table 2, we found that hydrophobic interactions occur only after the addition of aroma compounds, which were able to form relatively intact hydrophobic regions. These hydrophobic residues are crucial for stabilizing the complex formed by the sweet taste receptor and sucrose [23].

All 10 aroma-active compounds with sweetening effects could be stably bound to the sweet taste receptor-sucrose complex. Moreover, the addition of aroma compounds reduced the binding energy, formed new hydrogen bonds and hydrophobic interactions, and made sucrose bind more tightly to the sweet taste receptor. This result suggests that these aroma compounds enhance the interaction between sucrose and the sweet taste receptor, potentially contributing to the overall sweet perception in the tea infusions.

## Discussion

### Aroma attributes and structure of specific volatiles may contribute to enhanced sweetness

The perceived similarity between aroma and sweetness is crucial for sweetness enhancement [13]. In numerous flavour databases of volatiles (e.g., Perflavory Search and OdorantDB (Leibniz-lsb.de)), specific aroma attributes are frequently described as taste attributes, with “sweet” being a common aroma descriptor. Guichard et al. (2020) used a variety of multivariate statistical analyses to identify associations between aroma attributes and taste, revealing odors that enhance specific tastes [15]. The results showed that fruity, floral, or candy-like aromas are strongly correlated with sweetness. More than 100 sweetening volatiles have been identified, most of which are associated with fruit substrates such as bananas, oranges, and pomegranates [18,23,40]. Regarding volatile types, the primary aroma compounds for their sweetening effects are aldehydes, ketones, alcohols, and esters [13]. In this study, the ten sweetening aroma compounds identified in three high-sweet tea infusions (BT, WT, and YT) exhibited floral or fruity attributes and were closely associated with sweetness. Consequently, utilizing the perceived similarity and correlation between aromas and sweetness to select sweetening aroma compounds from numerous volatiles is highly effective, but its reliability requires further validation [13]. For example, *cis*-jasmone and 2-ethyl-1-hexanol, identified in this study as being associated with sweetness, exhibited no sweetening effect. Since perceptual interactions in aroma mixtures can either synergistically enhance or antagonistically inhibit a particular aroma attribute, understanding the flavor associations of a single aroma compound in isolation is insufficient for predicting its effects in complex aroma environments [41].

Previous studies have shown that specific chemical structures, such as aromatic groups, isoprenoid-like structures, and lactones, can enhance sweetness or produce sweet-like sensory characteristics in various food and beverage systems, including tea [13,15]. These findings suggest that sweetness enhancement may be linked to particular molecular patterns or functional groups within aroma compounds. For example, compounds that contain aromatic rings or ester linkages often exhibit pleasant, sweet odors and contribute to an overall perception of sweetness in beverages [13]. This result suggests that specific molecular structures could play a pivotal role in modulating sweetness perception in tea infusions. Additionally, when aroma compounds with similar chemical structures interact, they often exhibit synergistic effects, enhancing the volatility of the aroma or producing more pronounced olfactory experiences through chemical interactions between molecules [31,42]. Therefore, future research should further explore the interactions among sweetening aroma compounds with similar structures and their impact on sweetness.

Furthermore, it is important to note that the sweetness of tea infusions is not solely derived from aroma compounds but is also influenced by various taste compounds [43]. When different aroma compounds interact synergistically, they may affect the perception of aroma and influence the taste to varying degrees, thereby affecting the overall sweet sensation [44]. Hence, while much has been learned about the role of specific aroma compounds in enhancing sweetness, future studies should delve deeper into the complex interactions between aroma compounds and taste compounds. Understanding how these components interact to influence perceived sweetness will be essential for optimizing the flavor profile of tea beverages and developing strategies to enhance sweetness more controlled and targeted.

### Sweetening aroma compounds may have the potential to enhance sucrose binding to sweet taste receptors

The idea that sweetening aroma compounds can enhance the binding of sucrose to sweet taste receptors has attracted considerable attention in recent years, significantly as a means to reduce sugar content in foods while maintaining or enhancing perceived sweetness [23]. Human perception of sweetness begins with the interaction of sweet molecules with the T1R2/T1R3 heterodimeric receptors located on the apical surface of taste receptor cells in taste buds [13]. Recent research has shown that certain aroma compounds can modulate the interaction between sucrose and the sweet taste receptors (T1R2/T1R3) via various molecular interactions. Molecular docking and dynamics simulations have indicated that sweetening aroma compounds can form hydrogen bonds and hydrophobic interactions with the sweet taste receptors, thereby stabilizing the binding of sucrose and enhancing perceived sweetness [45]. For example, the (*E*)-citral in sweet orange has been shown to enhance the binding stability of sucrose to the T1R2/T1R3 receptor complex by modifying the hydrogen-bonding residues and significantly reducing the binding energy [13].

Furthermore, studies have identified specific amino acid residues (e.g., Ser144, Asp213, Asp278) as key interaction sites for aroma compounds, which can activate the sweet taste receptors and amplify the sweet signal [46]. In this study, we identified that *(E)*-*β*-damascenone, linalool, geraniol, dimethyl sulfide, (*E,E*)-2,4-heptadienal, (*E,Z*)-2,6-nonadienal, (*E*)-linalool oxide (furanoid), dihydroactinidiolide, *γ*-nonalactone, and (*E*)-*β*-ionone, previously shown to enhance sweetness in three high-sweet teas, also reduced the binding energy of sucrose to sweet taste receptors and formed new hydrogen bonds and hydrophobic interactions. In conclusion, sensory and molecular studies have well-established the ability of sweetening aroma compounds to enhance sucrose binding to sweet taste receptors. These findings offer valuable insights into the mechanisms underlying sweetness enhancement and present promising prospects for developing healthier food products with lower sugar content. Future research should focus on identifying additional aroma compounds with strong sweetening effects and exploring their mechanisms of action in greater detail. Furthermore, the effects of these compounds on different sweet taste receptor variants and their potential interactions with other taste modalities should be investigated.

## Conclusion

The present study found that aroma enhanced the sweet intensity of three tea infusions by over 24.0 %. Through GC-O-AT and sensory analysis, we identified 18 aroma-active compounds from 157 volatiles associated with sweetness. Among these, (*E*)-*β*-damascenone (apple-like), linalool (citrus-like), geraniol (citrus-like), dimethyl sulfide (corn-like), (*E,E*)-2,4-heptadienal (floral), (*E,Z*)-2,6-nonadienal (cucumber-like), (*E*)-linalool oxide (furanoid) (floral), dihydroactinidiolide (fruity), *γ*-nonalactone (coconut-like), and (*E*)-*β*-ionone (floral) were similar to sweet perception and significantly increased sweetness of sucrose by more than 7.5 % (*p* < 0.05). Molecular docking revealed that these aroma compounds bind stably to the sweet taste receptor-sucrose complex, reducing binding energy and forming new hydrogen bonds and hydrophobic interactions, potentially enhancing sucrose binding. This study suggests that aroma-active compounds in tea can naturally boost sweetness in tea infusion and may serve as food additives or flavour enhancers to improve sweetness perception.

Future research should explore the cross-modal interaction mechanisms between aroma and taste components. Integrating the interplay between aroma and taste with multi-dimensional sensory modalities (such as auditory, visual, and tactile senses) and brain neurophysiology can advance the study of multimodal flavour perception of food products. This integrated approach will enable more precise targeting of flavour modulation.

## Funding

This research was supported by the China Postdoctoral Science Foundation (2024M763608), the National Natural Science Foundation of China (32272368), China Central Public-Interest Scientific Institution Basal Research Fund (1630012025119), the Innovation Project for Chinese Academy of Agricultural Sciences (CAAS-ASTIP-TRI), the earmarked fund for CARS-19, and the National Key Laboratory for Tea Plant Germplasm Innovation and Resource Utilization (SQ2024SKL03104).

## CRediT authorship contribution statement

**Yuming Wei:** Conceptualization, Data curation, Formal analysis, Methodology, Writing – original draft. **Ya-Ya Yu:** Data curation, Investigation, Methodology. **Yuan-Chao Li:** Data curation, Investigation, Methodology, Resources. **Xiao-Yu Zhong:** Formal analysis, Methodology, Software. **Chun Zou:** Data curation, Investigation, Methodology. **Jingming Ning:** Visualization, Investigation, Supervision. **Wen-Jiang Dong:** Visualization, Investigation, Supervision. **Kegang Wu:** Visualization, Investigation, Supervision. **Yong-Quan Xu:** Funding acquisition, Project administration, Resources.

## Declaration of competing interest

The authors declare that they have no known competing financial interests or personal relationships that could have appeared to influence the work reported in this paper.
