# Supplementary material for: Aroma compounds with enhanced sweet perception in tea infusions: Screening, characterization, and sweetening mechanism
Source: J Adv Res. 2025 May 20;81:1–12. doi: 10.1016/j.jare.2025.05.044 (PMC12958201; doi:10.1016/j.jare.2025.05.044)

**Supplementary materials**

**Aroma compounds with enhanced sweet perception in tea infusions: Screening, characterization, and sweetening mechanism**

Yuming Wei^1^, Ya-Ya Yu^1^, Yuan-Chao Li^1^, Xiao-Yu Zhong^3^, Chun Zou^1^,

Jingming Ning^2^, Wen-Jiang Dong^4^, Kegang Wu^5^, Yong-Quan Xu^1*^

^1^ *Tea Research Institute Chinese Academy of Agricultural Sciences, Key Laboratory of Biology, Genetics and Breeding of Special Economic Animals and Plants, Ministry of Agriculture and Rural Affairs, Hangzhou 310008, China*

^2^ *National Key Laboratory for Tea Plant Germplasm Innovation and Resource Utilization, Hefei 230036, China*

^3^ *Zhejiang Minghuang Natural Food Development Co., Hangzhou 310030, China*

^4^ *Spice and Beverage Research Institute, Chinese Academy of Tropical Agricultural Sciences, Wanning 571533, China.*

^5^ *School of Chemical Engineering and Light Industry, Guangdong University of Technology, Guangzhou 510006, China.*

***Corresponding author at:**

Dr. Yong-Quan Xu, Email: [yqx33@126.com](mailto:yqx33@126.com), Tea Research Institute Chinese Academy of Agricultural Sciences, Key Laboratory of Biology, Genetics and Breeding of Special Economic Animals and Plants, Ministry of Agriculture and Rural Affairs, 9 South Meiling Road, Hangzhou 310008, China

**Running title:** Enhanced Sweet Perception Aroma Compounds in Tea Infusions

**Table S1** The contents of aroma compounds associated with sweetness in the tea samples based on the external standard quantitative method.

| Aroma compounds | m/z | Standard curve | Concentration  range (μg/L) | R² | Content (μg/L) | | | OT^a^ (μg/L) | OAV | | |
| --- | --- | --- | --- | --- | --- | --- | --- | --- | --- | --- | --- |
|  |  |  |  |  | BT | WT | YT |  | BT | WT | YT |
| 3-Methyl-2,4-nonanedione | 43 | y = 1.9570x - 0.0964 | 0.5 ~ 20.0 | 0.9991 | 0.9 | - | - | 0.046 | 19.6 | - | - |
| (*Z*)-3-Hexenal | 41 | y = 1.5056x - 0.1684 | 0.5 ~ 20.0 | 0.9921 | 1.9 | - | - | 0.12 | 15.6 | - | - |
| Dihydroactinidiolide | 111 | y = 1.2363x - 0.0407 | 0.2 ~ 120.0 | 0.9985 | 27.3 | 116.9 | 48.8 | 5 | 5.5 | 23.4 | 9.8 |
| Phenylacetaldehyde | 91 | y = 1.6973x - 0.2026 | 5.0 ~ 65.0 | 0.9994 | 56.8 | 13.1 | 48.4 | 5.2 | 10.9 | 2.5 | 9.3 |
| 1-Pentanol | 42 | y = 0.8064x + 0.0006 | 0.5 ~ 20.0 | 0.9997 | 3.9 | 4.6 | 13.1 | 0.67 | 5.9 | 6.9 | 19.6 |
| Dimethyl sulfide | 62 | y = 1.4513x - 0.3874 | 4.0 ~ 200.0 | 0.9982 | 7.4 | - | 18.7 | 0.3 | 24.6 | - | 62.2 |
| Linalool | 71 | y = 0.9982x - 0.3262 | 1.0 ~ 600.0 | 0.9977 | 541.7 | 53.9 | 46.9 | 0.58 | 934.0 | 92.9 | 80.9 |
| Geraniol | 69 | y = 1.232x - 0.5986 | 1.0 ~ 650.0 | 0.9956 | 117.6 | 162.0 | 609.7 | 1.1 | 107.0 | 147.3 | 554.3 |
| (*E*)-*β*-Ionone | 123 | y = 2.0738x + 0.0086 | 0.2 ~ 15.0 | 0.9962 | 4.1 | 6.7 | 9.6 | 0.021 | 194.9 | 319.0 | 455.3 |
| (*E,Z*)-2,6-Nonadienal | 69 | y = 0.7680x - 0.0347 | 0.2 ~ 15.0 | 0.9951 | 6.4 | 1.9 | 6.1 | 0.0045 | 1428.3 | 430.5 | 1361.8 |
| (*E,E*)-2,4-Heptadienal | 81 | y = 1.5734x - 0.0147 | 0.4 ~ 15.0 | 0.9947 | 8.2 | 1.5 | 50.7 | 0.032 | 255.4 | 47.1 | 1585.2 |
| (*E*)-Linalool oxide (furanoid) | 59 | y = 1.4356x - 0.6332 | 6.0 ~ 500.0 | 0.9943 | 406.6 | 256.8 | 198.0 | 190 | 2.1 | 1.4 | 1.0 |
| 2-Ethyl-1-hexanol | 57 | y = 0.4821x + 0.0568 | 1.0 ~ 80.0 | 0.9961 | 31.9 | 51.5 | 24.8 | 13 | 2.5 | 4.0 | 1.9 |
| *cis*-Jasmone | 164 | y = 0.5079x - 0.0816 | 1.0 ~ 40.0 | 0.9974 | 32.6 | 32.7 | 28.4 | 7 | 4.7 | 4.7 | 4.1 |
| *γ*-Nonalactone | 55 | y = 1.8732x - 0.1559 | 0.5 ~ 50.0 | 0.9937 | 3.8 | 37.6 | 6.0 | 9.7 | < 1.0 | 3.9 | < 1.0 |
| (*E*)-*β*-Damascenone | 69 | y = 2.4820x - 0.1675 | 0.2 ~ 12.0 | 0.9922 | 1.7 | - | - | 0.006 | 282.8 | - | - |
| 2-Heptanol | 70 | y = 2.1239x - 0.3934 | 0.5 ~ 150.0 | 0.9908 | 6.2 | - | - | 65.2 | < 1.0 | - | - |
| 2-Phenyl-2-butenal | 117 | y = 0.9214x - 0.0333 | 1.0 ~ 100.0 | 0.9934 | 33.5 | 2.0 | 4.9 | 32 | 1.0 | < 1.0 | < 1.0 |

^a^Odor threshold (OT) in water were obtained by referring to the study of Zhai et al., (2022), Wang et al., (2022), Yu et al., (2024), Flaig et al., (2020) and Leibniz-LSB@TUM odorant database (https://www.leibniz-lsb.de).

BT, black tea sample; WT, white tea sample; YT, yellow tea sample. “-” indicated no detected.

**Table S2** Samples for sweet intensity rating.

| No. | Aroma compounds added to sucrose solution | Concentration (μg/L) | | |
| --- | --- | --- | --- | --- |
|  |  | BT | WT | YT |
| 1 | 3-Methyl-2,4-nonanedione | 0.9 | - | - |
| 2 | (*Z*)-3-Hexenal | 1.9 | - | - |
| 3 | Dihydroactinidiolide | 27.3 | 116.9 | 48.8 |
| 4 | Phenylacetaldehyde | 56.8 | 13.2 | 48.4 |
| 5 | 1-Pentanol | 3.9 | 4.6 | 13.1 |
| 6 | Dimethyl sulfide | 7.4 | - | 18.7 |
| 7 | Linalool | 541.7 | 53.9 | 46.9 |
| 8 | Geraniol | 117.7 | 162.0 | 609.7 |
| 9 | (*E*)-*β*-Ionone | 4.1 | 6.7 | 9.6 |
| 10 | (*E,Z*)-2,6-Nonadienal | 6.4 | 1.9 | 6.1 |
| 11 | (*E,E*)-2,4-Heptadienal | 8.2 | 1.5 | 50.7 |
| 12 | (*E*)-Linalool oxide (furanoid) | 406.6 | 256.8 | 198.0 |
| 13 | 2-Ethyl-1-hexanol | 31.9 | 51.5 | 24.8 |
| 14 | *cis*-Jasmone | 32.6 | 32.7 | 28.4 |
| 15 | *γ*-Nonalactone | - | 37.6 | - |
| 16 | (*E*)-*β*-Damascenone | 1.7 | - | - |
| 17 | 2-Phenyl-2-butenal | 33.5 | - | - |

BT, black tea sample; WT, white tea sample; YT, yellow tea sample.

“-” indicated no added.

**Table S3** Volatile compounds and their odor description in black tea, white tea and yellow tea samples.

| No. | RI | CAS | Volatile compounds | Odor descriptions | OT^a^  (μg/L) | Relative content  (μg/L) | | | rOAV | | |
| --- | --- | --- | --- | --- | --- | --- | --- | --- | --- | --- | --- |
|  |  |  |  |  |  | BT | WT | YT | BT | WT | YT |
| **Aldehydes** | | | | | | | | | | | |
| 1 | 842 | 123-38-6 | propanal | - | - | - | 0.19 | - |  | - | - |
| 2 | 856 | 123-72-8 | butanal | malty, sweaty | 0.87 | 29.22 | 6.84 | 19.06 | 33.6 | 7.9 | 21.9 |
| 3 | 937 | 96-17-3 | 2-methylbutanal | malty | 1.5 | 106.98 | 20.95 | 53.06 | 71.3 | 14.0 | 35.4 |
| 4 | 941 | 590-86-3 | 3-methylbutanal | malty | 0.5 | 79.22 | 24.49 | 45.67 | 158.4 | 49.0 | 91.3 |
| 5 | 998 | 110-62-3 | pentanal | - | - | 5.06 | 4.51 | 20.33 | - | - | - |
| 6 | 1022 | 123-15-9 | 2-methyl-pentanal | - | - | 2.46 | - | - | - | - | - |
| 7 | 1102 | 66-25-1 | hexanal | green, grassy | 2.4 | 52.83 | 61.95 | 148.53 | 22.0 | 25.8 | 61.9 |
| 8 | 1115 | 497-03-0 | (*E*)-2-methyl-2-butenal | - | - | 1.01 | - | 1.17 | - | - | - |
| 9 | 1150 | 1576-87-0 | (*E*)-2-pentenal | - | - | - | - | 6.27 | - | - | - |
| 10 | 1154 | 6789-80-6 | (*Z*)-3-hexenal | green, grassy | 0.12 | 1.13 | - | - | 9.4 | - | - |
| 11 | 1175 | 623-36-9 | 2-methyl-2-pentenal | - | - | 1.00 | - | 4.05 | - | - | - |
| 12 | 1198 | 111-71-7 | heptanal | - | - | - | - | 8.89 | - | - | - |
| 13 | 1234 | 6728-26-3 | (*E*)-2-hexenal | green apple-like, bitter almond-like | 110 | 102.39 | 32.58 | 107.48 | 0.9 | 0.3 | 1.0 |
| 14 | 1253 | 6728-31-0 | (*Z*)-4-heptenal | oily, fatty | 0.0087 | 0.59 | - | 2.54 | 67.3 | - | 292.3 |
| 15 | 1294 | 124-13-0 | octanal | citrus-like, green | 3.4 | 1.68 | 3.60 | 4.05 | 0.5 | 1.1 | 1.2 |
| 16 | 1334 | 18829-55-5 | (*E*)-2-heptenal | - | - | 1.51 | - | 8.70 | - | - | - |
| 17 | 1398 | 124-19-6 | nonanal | citrus-like, soapy | 2.8 | 6.00 | 7.85 | 2.84 | 2.1 | 2.8 | 1.0 |
| 18 | 1414 | 142-83-6 | (*E,E*)-2,4-hexadienal | sweet, spicy, floral, citrus | 1.8 | 2.49 | - | 3.92 | 1.4 | - | 2.2 |
| 19 | 1437 | 2548-87-0 | (*E*)-2-octenal | - | - | 2.08 | 1.16 | 13.54 | - | - | - |
| 20 | 1473 | 4313-02-4 | (*E,Z*)-2,4-heptadienal | fatty, deep-fried | 94.8 | 2.80 | 0.15 | 38.97 | < 0.1 | < 0.1 | 0.4 |
| 21 | 1499 | 112-31-2 | decanal | soapy, citrus-like | 0.19 | - | 1.36 | 2.55 | - | 7.1 | 13.4 |
| 22 | 1499 | 4313-03-5 | (*E,E*)-2,4-heptadienal | fatty, floral | 0.032 | 12.71 | 2.23 | 79.67 | 397.3 | 69.5 | 2489.6 |
| 23 | 1534 | 100-52-7 | benzaldehyde | bitter almond-like | 150 | 57.72 | 80.37 | 62.69 | 0.4 | 0.5 | 0.4 |
| 24 | 1541 | 18829-56-6 | (*E*)-2-nonenal | - | - | 4.45 | 3.93 | 5.41 | - | - | - |
| 25 | 1594 | 557-48-2 | (*E,Z*)-2,6-nonadienal | cucumber-like | 0.0045 | 4.59 | 1.14 | 4.36 | 1019.8 | 253.5 | 968.8 |
| 26 | 1631 | 432-25-7 | *β*-cyclocitral | rose-like, sweet | 3 | 1.68 | 1.30 | 14.84 | 0.6 | 0.4 | 4.9 |
| 27 | 1636 | 620-23-5 | 3-methyl-benzaldehyde | - | - | 0.58 | - | - | - | - | - |
| 28 | 1650 | 3913-81-3 | (*E*)-2-decenal | - | - | - | - | 1.00 | - | - | - |
| 29 | 1655 | 116-26-7 | safranal | - | - | - | 1.87 | 2.24 | - | - | - |
| 30 | 1657 | 122-78-1 | phenylacetaldehyde | honey-like, floral | 5.2 | 94.34 | 20.29 | 80.08 | 18.1 | 3.9 | 15.4 |
| 31 | 1689 | 106-26-3 | neral | citrus-like, soapy | 100 | 3.48 | 4.56 | 11.71 | < 0.1 | < 0.1 | 0.1 |
| 32 | 1710 | 5910-87-2 | (*E,E*)-2,4-nonadienal | fatty, floral | 0.046 | - | - | 4.34 | - | - | 94.2 |
| 33 | 1740 | 141-27-5 | geranial | citrus-like | 32 | 4.33 | 3.67 | 22.95 | 0.1 | 0.1 | 0.7 |
| 34 | 1810 | 2111-75-3 | perillaldehyde | - | - | - | - | 1.46 | - | - | - |
| 35 | 1821 | 25152-84-5 | (*E,E*)-2,4-decadienal | fatty, fruity | 0.027 | - | - | 4.59 | - | - | 170.0 |
| 36 | 1948 | 4411-89-6 | 2-phenyl-2-butenal | honey-like, sweet | 42 | 30.52 | 1.50 | 4.18 | 0.7 | < 0.1 | 0.1 |
| 37 | 2020 | 101-39-3 | *α*-methylcinnamaldehyde | - | - | - | 0.96 | - | - | - | - |
| 38 | 2062 | 14371-10-9 | cinnamaldehyde | - | - | 0.42 | - | - | - | 7.9 | - |
| 39 | 2090 | 21834-92-4 | 5-methyl-2-phenyl-2-hexenal | - | - | 1.35 | - | - | - | 14.0 | - |
| **Alcohols** | | | | | | | | | | | |
| 1 | 1185 | 616-25-1 | 1-penten-3-ol | - | - | 9.68 | 0.34 | 8.82 | - | - | - |
| 2 | 1226 | 137-32-6 | 2-methylbutan-1-ol | - | - | - | - | 0.49 | - | - | - |
| 3 | 1264 | 71-41-0 | 1-pentanol | fruity, ethereal | 0.67 | 3.19 | 3.74 | 10.58 | 4.8 | 5.6 | 15.8 |
| 4 | 1328 | 543-49-7 | 2-heptanol | coconut-like, sweet | 65.2 | 9.18 | - | - | 0.1 | - | - |
| 5 | 1331 | 1576-95-0 | *cis*-2-penten-1-ol | - | - | 4.03 | - | 4.76 | - | - | - |
| 6 | 1362 | 111-27-3 | 1-hexanol | green, fruity | 590 | 42.24 | 4.88 | 16.87 | 0.1 | < 0.1 | < 0.1 |
| 7 | 1372 | 928-97-2 | (*E*)-3-hexen-1-ol | - | - | 0.57 | - | 1.30 | - | - | - |
| 8 | 1392 | 928-96-1 | (*Z*)-3-hexen-1-ol | green, grassy | 3.9 | 31.32 | 6.18 | 56.47 | 8.0 | 1.6 | 14.5 |
| 9 | 1412 | 928-95-0 | (*E*)-2-hexen-1-ol | green, grassy | 230 | 21.91 | - | 5.96 | 0.1 | - | - |
| 10 | 1453 | 3391-86-4 | 1-octen-3-ol | mushroom-like | 45 | 15.57 | 8.29 | 14.33 | 0.3 | 0.2 | 0.3 |
| 11 | 1458 | 111-70-6 | 1-heptanol | fruity, soapy | 5.4 | 1.51 | 1.23 | 6.78 | 0.3 | 0.2 | 1.3 |
| 12 | 1479 | 34995-77-2 | (*E*)-linalool oxide (furanoid) | floral | 190 | 577.34 | 362.31 | 277.86 | 3.0 | 1.9 | 1.5 |
| 13 | 1490 | 104-76-7 | 2-ethyl-1-hexanol | sweet, ethereal, floral | 13 | 15.94 | 25.39 | 12.51 | 1.2 | 2.0 | 1.0 |
| 14 | 1556 | 78-70-6 | linalool | citrus-like, floral | 0.58 | 537.45 | 50.53 | 43.57 | 926.6 | 87.1 | 75.1 |
| 15 | 1560 | 111-87-5 | 1-octanol | green, citrus-like | 2.7 | 1.30 | 1.02 | 3.81 | 0.5 | 0.4 | 1.4 |
| 16 | 1608 | 562-74-3 | terpinen-4-ol | - | - | 3.55 | 1.81 | - | - | - | - |
| 17 | 1611 | 29957-43-5 | 3,7-dimethyl-1,5,7-octatrien-3-ol | - | - | 26.35 | - | 81.76 | - | - | - |
| 18 | 1616 | 18409-17-1 | (*E*)-2-octen-1-ol | - | - | 1.15 | - | 1.06 | - | - | - |
| 19 | 1642 | 89-78-1 | menthol | mint-like | 130 | - | 2.10 | 0.96 | - | < 0.1 | < 0.1 |
| 20 | 1659 | 143-08-8 | 1-nonanol | soapy, fruity | 0.9 | 2.55 | 1.55 | 3.62 | 2.8 | 1.7 | 4.0 |
| 21 | 1702 | 98-55-5 | *α*-terpineol | floral, citrus-like | 1200 | 80.11 | 29.30 | 10.07 | 0.1 | < 0.1 | < 0.1 |
| 22 | 1743 | 14009-71-3 | (*Z*)-linalool oxide (pyranoid) | - | - | - | 10.18 | 18.42 | - | - | - |
| 23 | 1767 | 39028-58-5 | (*E*)-linalool oxide (pyranoid) | woody | 3000 | 90.44 | 49.03 | 28.13 | < 0.1 | < 0.1 | < 0.1 |
| 24 | 1802 | 106-25-2 | nerol | citrus-like, floral | 49 | 18.06 | 6.70 | 16.72 | 0.4 | 0.1 | 0.3 |
| 25 | 1820 | 98-85-1 | phenethyl alcohol | - | - | - | 0.78 | - | - | - | - |
| 26 | 1849 | 106-24-1 | geraniol | rose-like, citrus-like | 1.1 | 138.98 | 193.66 | 745.20 | 126.3 | 176.1 | 677.5 |
| 27 | 1872 | 90-05-1 | guaiacol | - | - | 0.75 | 0.30 | - | - | - | - |
| 28 | 1886 | 100-51-6 | benzyl alcohol | bitter almond-like, floral | 62 | 19.01 | 30.96 | 27.46 | 0.3 | 0.5 | 0.4 |
| 29 | 1923 | 60-12-8 | 2-phenylethanol | floral, honey-like | 140 | 108.85 | 103.01 | 171.28 | 0.8 | 0.7 | 1.2 |
| 30 | 2015 | 108-95-2 | phenol | - | - | 15.77 | 14.46 | 11.14 | - | - | - |
| 31 | 2039 | 40716-66-3 | (*E*)-nerolidol | - | - | 0.84 | - | 4.94 | - | - | - |
| 32 | 2091 | 108-39-4 | *m*-cresol | - | - | - | 0.48 | - | - | - | - |
| 33 | 2112 | 536-60-7 | *p*-cymen-7-ol | - | - | - | - | 2.18 | - | - | - |
| 34 | 2181 | 97-53-0 | eugenol | - | - | 2.41 | 0.54 | 0.45 | - | - | - |
| 35 | 2185 | 620-17-7 | 3-ethylphenol | - | - | 0.32 | - | 0.11 |  |  |  |
| 36 | 2212 | 7786-61-0 | 2-methoxy-4-vinylphenol | smoky, clove-like | 12 | 0.91 | - | 0.99 | 0.1 | - | 0.1 |
| 37 | 2366 | 5932-68-3 | (*E*)-isoeugenol | - | - | 1.69 | - | - | - | - | - |
| 38 | 2406 | 2628-17-3 | 4-vinylphenol | - | - | 5.18 | 1.91 | 2.54 | - | - | - |
| **Ketones** | | | | | | | | | | | |
| 1 | 927 | 78-93-3 | 2-butanone | - | - | 1.55 | 4.86 | 1.02 | - | - | - |
| 2 | 996 | 431-03-8 | 2,3-butanedione | - | - | 0.98 | - | 0.55 | - | - | - |
| 3 | 1039 | 1629-58-9 | 1-penten-3-one | - | - | 0.53 | 0.20 | 3.23 | - | - | - |
| 4 | 1081 | 600-14-6 | 2,3-pentanedione | - | - | - | 0.07 | 0.37 | - | - | - |
| 5 | 1151 | 141-79-7 | mesityl oxide | - | - | 1.81 | 1.18 | 8.20 | - | - | - |
| 6 | 1196 | 110-43-0 | 2-heptanone | - | - | 5.49 | 5.10 | 12.62 | - | - | - |
| 7 | 1291 | 111-13-7 | 2-octanone | - | - | - | - | 1.61 | - | - | - |
| 8 | 1320 | 116-09-6 | hydroxyacetone | - | - | - | 0.16 | 0.99 | - | - | - |
| 9 | 1333 | 585-25-1 | 2,3-octanedione | - | - | 0.71 | 1.61 | 2.35 | - | - | - |
| 10 | 1348 | 110-93-0 | 6-methyl-5-hepten-2-one | - | - | 8.22 | 19.05 | 69.36 | - | - | - |
| 11 | 1415 | 18402-82-9 | (*E*)-3-octen-2-one | floral, spicy | 300 | 3.49 | 1.12 | 12.59 | < 0.1 | < 0.1 | < 0.1 |
| 12 | 1526 | 38284-27-4 | 3,5-octadien-2-one | - | - | 33.36 | 2.52 | 59.56 | - | - | - |
| 13 | 1580 | 30086-02-3 | (*E,E*)-3,5-octadien-2-one | musty, fatty | 14 | 18.98 | 9.15 | 22.92 | 1.4 | 0.7 | 1.6 |
| 14 | 1603 | 78-59-1 | isophorone | camphor-like | 110000 | - | 3.09 | 0.69 | - | < 0.1 | < 0.1 |
| 15 | 1646 | 96-48-0 | *γ*-butyrolactone | sweet, caramel, creamy | 50 | 0.60 | 0.69 | 1.73 | < 0.1 | < 0.1 | < 0.1 |
| 16 | 1664 | 98-86-2 | 1-phenylethanone | bitter almond-like | 65 | 3.83 | 10.86 | 4.08 | 0.1 | 0.2 | 0.1 |
| 17 | 1704 | 1125-21-9 | 4-ketoisophorone | - | - | 0.42 | 2.46 | 2.01 | - | - | - |
| 18 | 1719 | 695-06-7 | *γ*-hexalactone | coconut-like, fruity | 12500 | 1.02 | 3.89 | 4.60 | - | - | - |
| 19 | 1730 | 113486-29-6 | 3-methyl-2,4-nonanedione | rose-like, fruity | 0.046 | 0.81 | - | - | 17.6 | - | - |
| 20 | 1792 | 122-00-9 | *p*-methyl-acetophenone | - | - | 1.54 | 3.29 | 2.29 | - | - | - |
| 21 | 1833 | 23726-93-4 | (*E*)-*β*-damascenone | sweet, apple-like | 0.006 | 2.54 | - | - | 422.8 | - | - |
| 22 | 1862 | 127-41-3 | *α*-ionone | - | - | 1.92 | 4.90 | 4.21 | - | - | - |
| 23 | 1952 | 79-77-6 | (*E*)-*β*-ionone | floral, violet-like | 0.021 | 8.58 | 13.98 | 19.92 | 408.4 | 665.6 | 948.4 |
| 24 | 1959 | 488-10-8 | *cis*-jasmone | woody, herbal, floral | 7 | 15.73 | 15.79 | 13.61 | 2.2 | 2.3 | 1.9 |
| 25 | 2008 | 23267-57-4 | *β*-ionone epoxide | - | - | 19.16 | 116.99 | - | - | - | - |
| **Heterocycles** | | | | | | | | | | | |
| 1 | 907 | 534-22-5 | 2-methyl-furan | - | - | - | - | 1.21 | - | - | - |
| 2 | 973 | 3208-16-0 | 2-ethyl-furan | - | - | 4.47 | 0.77 | 11.47 | - | - | - |
| 3 | 1197 | 617-92-5 | 1-ethyl-1H-pyrrole | - | - | 4.35 | - | - | - | - | - |
| 4 | 1236 | 3777-69-3 | 2-pentylfuran | fruity, green | 19 | 1.11 | 2.49 | 1.58 | 0.1 | 0.1 | 0.1 |
| 5 | 1281 | 109-08-0 | 2-methylpyrazine | - | - | - | 2.06 | - | - | - | - |
| 6 | 1335 | 108-50-9 | 2,6-dimethylpyrazine | - | - | - | 2.36 | - | - | - | - |
| 7 | 1347 | 13925-00-3 | ethyl-pyrazine | - | - | - | 1.03 | - | - | - | - |
| 8 | 1399 | 13360-64-0 | 2-ethyl-5-methyl-pyrazine | nutty, roasty | 13 | - | 2.63 | - | < 0.1 | 0.2 | < 0.1 |
| 9 | 1413 | 14667-55-1 | 2,3,5-trimethylpyrazine | - | - | - | 0.85 | - | - | - | - |
| 10 | 1441 | 13360-65-1 | 2-ethyl-3,6-dimethylpyrazine | - | - | - | 0.33 | - | - | - | - |
| 11 | 1452 | 13925-07-0 | 2-ethyl-3,5-dimethylpyrazine | - | - | - | 1.64 | - | - | - | - |
| 12 | 1475 | 98-01-1 | 2-furfural | - | - | 2.40 | 0.72 | - | - | - | - |
| 13 | 1514 | 1192-62-7 | 2-acetylfuran | caramel, cocoa | 15025 | 1.20 | - | - | < 0.1 | - | - |
| 14 | 1584 | 3194-15-8 | 2-propionylfuran | - | - | - | 0.29 | - | - | - | - |
| 15 | 1622 | 2167-14-8 | 1-ethyl-2-formyl-1H-pyrrole | roasty, smoky | 65000 | 94.57 | 14.55 | 18.60 | < 0.1 | < 0.1 | < 0.1 |
| 16 | 1624 | 1193-79-9 | 2-acetyl-5-methylfurane | roasty | 30000 | - | 0.90 | - | - | < 0.1 | - |
| 17 | 1665 | 98-00-0 | furfuryl alcohol | - | - | 1.75 | 1.89 | 0.86 | - | - | - |
| 18 | 1985 | 1072-83-9 | 2-acetyl-1H-pyrrole | - | - | - | 4.16 | - | - | - | - |
| 19 | 2474 | 120-72-9 | indole | floral | 11 | 2.72 | 1.69 | 75.56 | 0.2 | 0.2 | 6.9 |
| 20 | 2520 | 83-34-1 | skatole | - | - | 1.52 | 1.14 | - | - | - | - |
| **Esters** | | | | | | | | | | | |
| 1 | 1325 | 3681-82-1 | (*E*)-3-hexenyl acetate | - | - | - | - | 3.05 | - | - | - |
| 2 | 1463 | 16491-36-4 | (*Z*)-3-hexenyl butyrate | - | - | - | - | 4.67 | - | - | - |
| 3 | 1684 | 1073-11-6 | lavender lactone | - | - | - | 1.88 | 0.43 | - | - | - |
| 4 | 1739 | 140-11-4 | benzyl acetate | sweet, fruity | 364 | - | 3.03 | - | - | < 0.1 | - |
| 5 | 1793 | 119-36-8 | methyl salicylate | mint-like | 40 | 244.45 | 108.73 | 94.12 | 6.1 | 2.7 | 2.4 |
| 6 | 1827 | 103-45-7 | 2-phenylethyl acetate | honey-like, floral | 360 | 1.90 | 11.31 | 11.62 | < 0.1 | < 0.1 | < 0.1 |
| 7 | 1936 | 104-50-7 | *γ*-octalactone | coconut-like | 6.5 | 6.70 | 7.43 | - | 1.0 | 1.1 | - |
| 8 | 1976 | 103-52-6 | phenethyl butyrate | - | - | - | - | 7.95 | - | - | - |
| 9 | 1984 | 24817-51-4 | 2-phenylethyl-2-methylbutyrat | - | - | - | - | 6.89 | - | - | - |
| 10 | 1989 | 698-76-0 | *δ*-octalactone | coconut-like | 100 | 0.96 | 1.76 | 6.10 | < 0.1 | < 0.1 | 0.1 |
| 11 | 2049 | 104-61-0 | *γ-*nonalactone | coconut-like | 9.7 | 5.56 | 68.76 | 9.71 | 0.6 | 7.1 | 1.0 |
| 12 | 2217 | 705-86-2 | *δ*-decalactone | coconut-like | 31 | 5.92 | - | 41.55 | 0.2 | - | 1.3 |
| 13 | 2285 | 25524-95-2 | jasmine lactone | creamy, coconut-like | 2000 | 11.05 | - | 356.50 | < 0.1 | - | 0.2 |
| 14 | 2353 | 1211-29-6 | methyl jasmonate | floral | 70 | 5.00 | 1.93 | 43.36 | 0.1 | < 0.1 | 0.6 |
| 15 | 2388 | 17092-92-1 | dihydroactinidolide | fruit, woody | 5 | 33.29 | 144.09 | 59.89 | 6.7 | 28.8 | 12.0 |
| 16 | 2494 | 91-64-5 | coumarin | woodruff-like, almond paste-like | 11 | - | 6.50 | 4.25 | - | 0.6 | 0.4 |
| **Acids** | | | | | | | | | | | |
| 1 | 1631 | 107-92-6 | butanoic acid | - | - | - | 0.22 | - | - | - | - |
| 2 | 1846 | 142-62-1 | hexanoic acid | - | - | 5.01 | 15.01 | - | - | - | - |
| 3 | 1953 | 111-14-8 | heptanoic acid | - | - | - | 7.46 | 2.87 | - | - | - |
| 4 | 2059 | 124-07-2 | octanoic acid | carrot-like, musty | 3000 | 5.95 | 7.19 | 9.42 | < 0.1 | < 0.1 | < 0.1 |
| 5 | 2166 | 112-05-0 | nonanoic acid | - | - | - | 32.26 | 5.76 | - | - | - |
| 6 | 2273 | 334-48-5 | *n*-decanoic acid | - | - | - | - | 1.94 | - | - | - |
| 7 | 2342 | 4698-08-2 | geranic acid | - | - | 12.38 | 43.15 | 9.05 | - | - | - |
| 8 | 2905 | 57-10-3 | *n*-hexadecanoic acid | - | - | - | 0.69 | - | - | - | - |
| **Terpenes** | | | | | | | | | | | |
| 1 | 1166 | 123-35-3 | *β*-myrcene | peppery, spicy | 1.2 | 6.58 | 2.44 | 11.96 | 5.5 | 2.0 | 10.0 |
| 2 | 1199 | 138-86-3 | limonene | citrus-like | 13 | 4.47 | - | 5.19 | 0.3 | - | 0.4 |
| 3 | 1255 | 3779-61-1 | (*E*)-*β*-ocimene | sweet, herbal | 97 | 2.22 | - | 8.18 | < 0.1 | - | 0.1 |
| **Others** | | | | | | | | | | | |
| 1 | 817 | 75-18-3 | dimethyl sulfide | corn-like | 0.3 | 6.85 | - | 22.86 | 22.8 | - | 76.2 |
| 2 | 1057 | 108-88-3 | toluene | - | - | 7.93 | 0.70 | 10.63 | - | - | - |
| 3 | 1092 | 624-92-0 | dimethyl disulfide | - | - | 0.27 | - | - | - | - | - |
| 4 | 1149 | 106-42-3 | *p*-xylene | - | - | - | 0.08 | 0.38 | - | - | - |
| 5 | 1285 | 95-63-6 | 1,2,4-trimethyl-benzene | - | - | - | 0.02 | 1.20 | - | - | - |
| 6 | 1757 | 91-20-3 | naphthalene | - | - | - | 0.17 | - | - | - | - |
| 7 | 1947 | 140-29-4 | phenyl acetonitrile | - | - | 12.12 | 0.45 | 188.90 | - | - | - |
| 8 | 1979 | 95-16-9 | benzothiazole | - | - | 0.37 | 0.07 | - | - | - | - |

^a^Odor threshold (OT) in water were obtained by referring to the study of Zhai et al., (2022), Wang et al., (2022), Yu et al., (2024), Flaig et al., (2020) and Leibniz-LSB@TUM odorant database (https://www.leibniz-lsb.de).

**Figure S1.** T1R2-T1R3 dimer, red areas indicate VFD docking pockets.


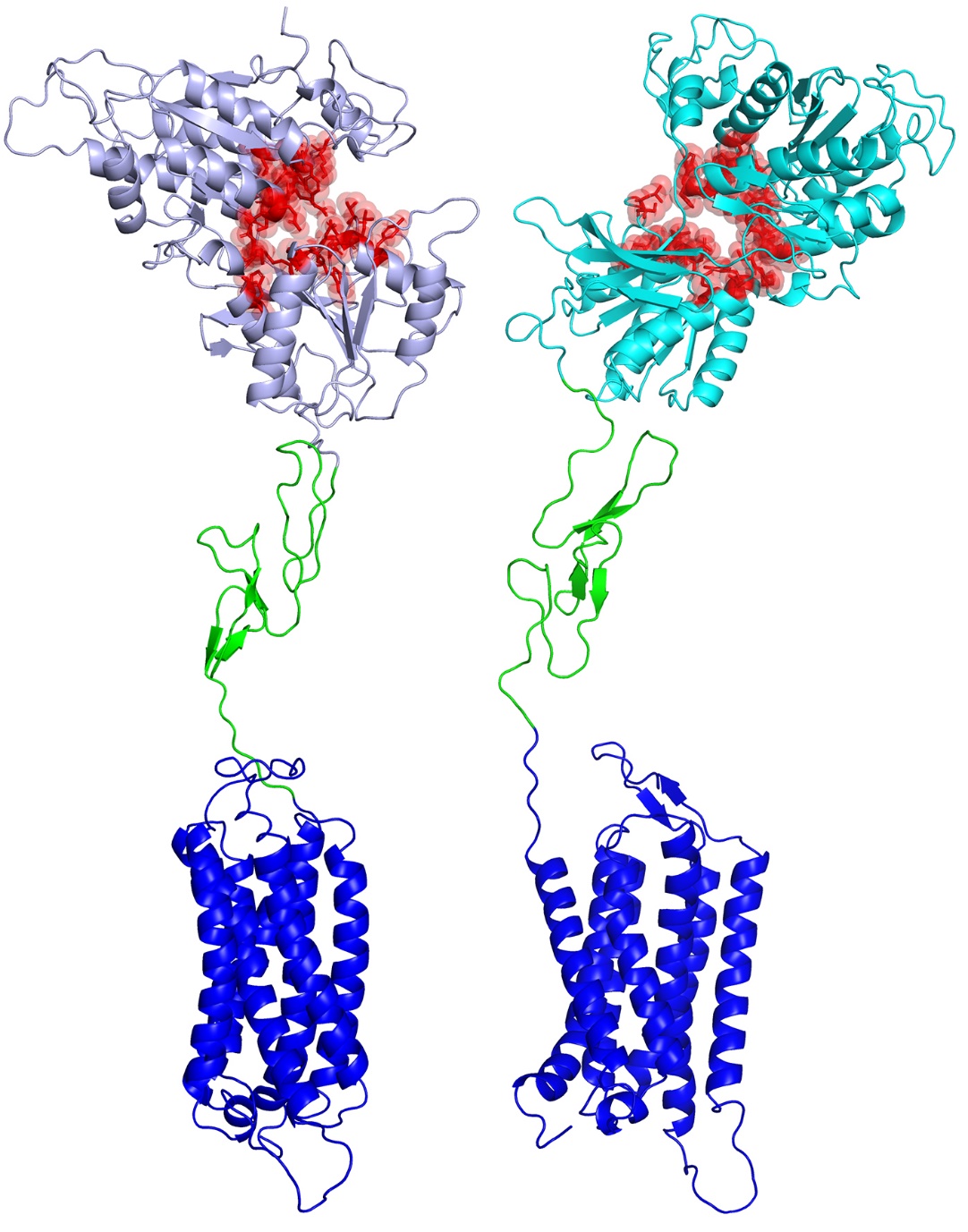

Supplement: Supplementary Data 1 [file mmc1.docx]
